# Effects of dopaminergic treatment on inhibitory control differ across Hoehn and Yahr stages of Parkinson’s disease

**DOI:** 10.1093/braincomms/fcad350

**Published:** 2023-12-20

**Authors:** Giovanni Mirabella, Andrea Pilotto, Andrea Rizzardi, Martina Montalti, Enrica Olivola, Cinzia Zatti, Veronica Di Caprio, Elisabetta Ferrari, Nicola Modugno, Alessandro Padovani

**Affiliations:** Department of Clinical and Experimental Sciences, University of Brescia, 25123 Brescia, BS, Italy; IRCCS Neuromed, 86077 Pozzilli, IS, Italy; Department of Clinical and Experimental Sciences, Neurology Unit, University of Brescia, 25123 Brescia, BS, Italy; Laboratory of Digital Neurology and Biosensors, University of Brescia, 25123 Brescia, BS, Italy; Department of Continuity of Care and Frailty, Neurology Unit, ASST Spedali Civili Brescia Hospital, 25123 Brescia, BS, Italy; Department of Clinical and Experimental Sciences, Neurology Unit, University of Brescia, 25123 Brescia, BS, Italy; Laboratory of Digital Neurology and Biosensors, University of Brescia, 25123 Brescia, BS, Italy; Department of Clinical and Experimental Sciences, University of Brescia, 25123 Brescia, BS, Italy; IRCCS Neuromed, 86077 Pozzilli, IS, Italy; Department of Clinical and Experimental Sciences, Neurology Unit, University of Brescia, 25123 Brescia, BS, Italy; Laboratory of Digital Neurology and Biosensors, University of Brescia, 25123 Brescia, BS, Italy; Department of Continuity of Care and Frailty, Neurology Unit, ASST Spedali Civili Brescia Hospital, 25123 Brescia, BS, Italy; IRCCS Neuromed, 86077 Pozzilli, IS, Italy; Department of Clinical and Experimental Sciences, Neurology Unit, University of Brescia, 25123 Brescia, BS, Italy; IRCCS Neuromed, 86077 Pozzilli, IS, Italy; Department of Clinical and Experimental Sciences, Neurology Unit, University of Brescia, 25123 Brescia, BS, Italy; Laboratory of Digital Neurology and Biosensors, University of Brescia, 25123 Brescia, BS, Italy; Department of Continuity of Care and Frailty, Neurology Unit, ASST Spedali Civili Brescia Hospital, 25123 Brescia, BS, Italy

**Keywords:** Parkinson’s disease, dopaminergic medication, reactive inhibition, proactive inhibition, overdose dopamine hypothesis

## Abstract

Motor inhibitory control, a core component of cognitive control, is impaired in Parkinson’s disease, dramatically impacting patients’ abilities to implement goal-oriented adaptive strategies. A progressive loss of the midbrain’s dopamine neurons characterizes Parkinson’s disease and causes motor features responsive to dopaminergic treatments. Although such treatments restore motor symptoms, their impact on response inhibition is controversial. Most studies failed to show any effect of dopaminergic medicaments, although three studies found that these drugs selectively improved inhibitory control in early-stage patients. Importantly, all previous studies assessed only one domain of motor inhibition, i.e. reactive inhibition (the ability to react to a stop signal). The other domain, i.e. proactive inhibition (the ability to modulate reactive inhibition pre-emptively according to the current context), was utterly neglected. To re-examine this issue, we recruited cognitively unimpaired Parkinson’s patients under dopaminergic treatment in the early (Hoehn and Yahr, 1–1.5, *n* = 20), intermediate (Hoehn and Yahr 2, *n* = 20), and moderate/advanced (Hoehn and Yahr, 2.5–3, *n* = 20) stages of the disease. Using a cross-sectional study design, we compared their performance on a simple reaction-time task and a stop-signal task randomly performed twice on dopaminergic medication (ON) and after medication withdrawal (OFF). Normative data were collected on 30 healthy controls. Results suggest that medication effects are stage-dependent. In Hoehn and Yahr 1–1.5 patients, drugs selectively impair reactive inhibition, leaving proactive inhibition unaffected. In the ON state, Hoehn and Yahr two patients experienced impaired proactive inhibition, whereas reactive inhibition is no longer affected, as it deteriorates even during the OFF state. By contrast, Hoehn and Yahr 2.5–3 patients exhibited less efficient reactive and proactive inhibition in the OFF state, and medication slightly improved proactive inhibition. This evidence aligns with the dopamine overdose hypothesis, indicating that drug administration may overdose intact dopamine circuitry in the earliest stages, impairing associated cognitive functions. In later stages, the progressive degeneration of dopaminergic neurons prevents the overdose and can exert some beneficial effects. Thus, our findings suggest that inhibitory control assessment might help tailor pharmacological therapy across the disease stage to enhance Parkinson’s disease patients’ quality of life by minimizing the hampering of inhibitory control and maximizing the reduction of motor symptoms.

## Teaser

Mirabella *et al*. examined the impact of dopaminergic treatment (DT) on motor inhibition in Parkinson’s patients. They found that DT’s effects vary depending on the disease stage. DT hinders inhibitory control in early-stage patients. These results emphasize the importance of carefully adjusting DT in the initial stages of the disease.

## Graphical Abstract

**Graphical Abstract:**
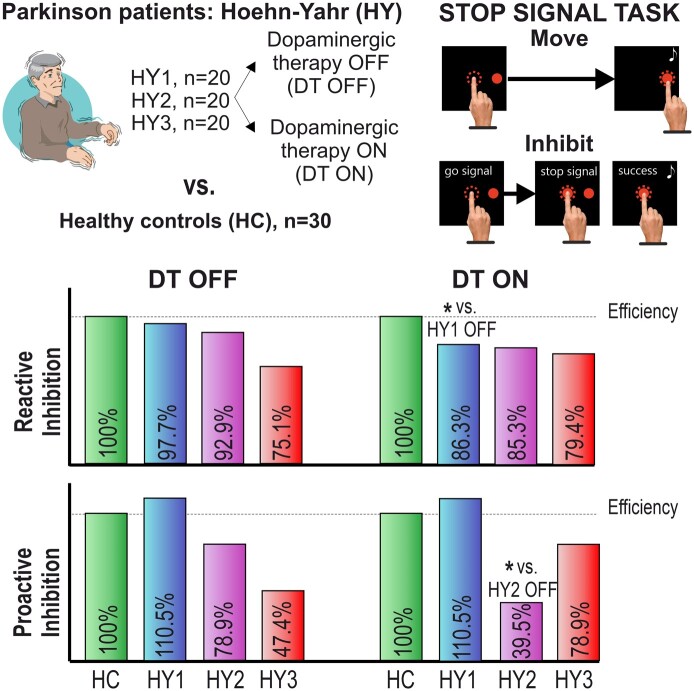

## Introduction

Inhibitory control is a pillar of cognitive control, enabling the execution of adaptive and flexible behaviours.^1^ In Parkinson’s disease, this executive function is compromised, significantly impacting patients’ ability to perform goal-oriented/voluntary actions.^2-4^ Parkinson’s disease is associated with a loss of the midbrain’s dopamine neurons, resulting in motor symptoms that can be alleviated through pharmacological dopaminergic treatment (DT), which includes levodopa (L-dopa), dopamine catabolism inhibitors, and dopamine receptor agonists (DA). Paradoxically, despite dopamine’s known involvement in response inhibition,^5-7^ the effects of DT on inhibitory control remain largely unclear. Given that response inhibition is a pivotal component in decision-making and action control,^8^ and considering that individuals with Parkinson’s disease already experience its impairment, it becomes imperative to enhance our comprehension of the impact of DT on this fundamental executive function. This deeper understanding will enable the customization of medication regimens to better align with the specific needs of patients. Building on our recent evidence indicating differences in inhibitory control deficits between early^3^ and later stages^4^ of Parkinson’s disease, our current study seeks to contrast the performance of response inhibition in Parkinson’s disease patients at distinct stages under pharmacological treatment (ON) and after an overnight wash-out of at least 12 h (OFF). To measure the impact of DT on inhibition proficiency, we used the stop-signal task (SST), a gold standard model of inhibitory control.^9^

To obtain a current perspective of the research, we conducted a systematic review by searching PubMed and Scopus for studies investigating the effects of DT on response inhibition in Parkinson’s disease in ON versus OFF therapy. We identified nine studies (Supplementary material, Paragraph 1, Supplementary Fig. 1, Supplementary Table 1). All focused on reactive inhibition (the ability to stop a response at the presentation of a stop-signal abruptly), neglecting the other domain of inhibitory control, i.e. proactive inhibition (the ability to modulate reactive inhibition in advance according to one’s goal).^10^ Eight studies addressed the effect of DT, and one addressed the effect of DA. Six studies found no effect of DT/DA, while three found that DT improved reactive inhibition. Several reasons can explain such incongruences. First, four studies have <15 participants, which is unlikely to compensate for the intrinsic variability of patients’ performance. Second, in two studies,^11,12^ the assumptions of the horse-race model,^13^ i.e. the independence between the stop- and the go-processes,^9^ needed to compute the measure of reactive inhibition, i.e. the stop signal reaction time (SSRT, the time it takes to suppress an ongoing action), were violated, calling into question the interpretability of the results (Supplementary material, Paragraph 5, Supplementary Table 5 for further details). Third, in three studies, the counterbalancing was not applied; thus, a learning effect is likely to occur, weakening the results’ value. Fourth, all studies’ samples were composed of patients at different stages of Parkinson’s disease and with a wide range of disease duration. This poses a severe limitation to the results’ interpretation because, first, the inhibitory deficit changes along the course of the disease.^3,4^ Second, the degeneration of dopaminergic neurons has a well-recognized temporal pattern. In the early stages, neurons of the substantia nigra pars compacta projecting to the dorsal striatum are much more affected than those directed to the ventral striatum,^14^ and neurons of the ventral tegmental area are relatively spared.^15^ According to the dopamine overdose hypothesis (DOH),^16^ in the early stages of Parkinson’s disease, DT improves motor and cognitive functions relying on the depleted dorsolateral striatal circuits but overdoses the dopaminergic neurons in the relatively unaffected circuitry of the ventral striatum and associated prefrontal areas, impairing cognitive functions depending on these networks. In the later stages of Parkinson’s disease, the DOH predicts that DT should not overdose dopaminergic circuitry of the ventral striatum because of their degeneration and can even improve the cognitive function it hampered in the previous stages. In conclusion, the effects of DT on motor inhibition remain poorly understood.

To address these limitations, we conducted a novel study where we assessed both reactive and proactive inhibition in three groups of Parkinson’s disease patients categorized as early [Hoehn and Yahr (H&Y) 1–1.5], moderate (H&Y 2) and moderate-advanced (H&Y 2.5–3) stages, under ON and OFF conditions. We used the H&Y staging scores because they correlate with patients’ level of disability, quality of life, and dopaminergic loss.^17^ We hypothesized that DT would influence motor inhibition in the earliest stages but not in the more advanced ones when dopaminergic cells are too few to be overdosed. Given that the only previous study with a relatively homogeneous cohort of Parkinson’s disease patients in the early stages (H&Y 1–2) found that DT administration improved reactive inhibition, we expected to find (i) a significant amelioration of reactive motor inhibition in the H&Y 1–1.5 group in ON compared with OFF condition and (ii) no ON/OFF change of the SSRT length in the H&Y 2.5–3.

## Materials and methods

### Participants

Seventy idiopathic established Parkinson’s disease patients^18^ of Caucasian ethnicity at H&Y Stages 1–3 were recruited from the outpatients of the IRCCS Neuromed Hospital, Pozzilli, Italy, and the Movement Disorder clinic at ASST Spedali Civili Hospital of Brescia, Italy, between September 2019 and October 2022. All patients were under a stable treatment regimen for at least 6 months with levodopa (L-dopa) and/or DA and/or inhibitors of dopamine catabolism pharmacotherapy. Participants had normal or corrected-to-normal vision and were all right-handed but one as assessed by the Edinburgh Handedness Inventory.^19^ Exclusion criteria were (i) the presence of neurological disease besides Parkinson’s disease; (ii) mini-mental state examination (MMSE) <24 as values below such threshold indicate possible cognitive impairment;^20^ (iii) severe tremor and/or presence of severe sensory deficits; (iv) symptoms of impulse control disorder (ICD)^21^ assessed via the Questionnaire for Impulsive–Compulsive Disorders in Parkinson’s Disease–Rating Scale, given that patients with ICD are faster at stopping;^22^ (v) major depressive disorders requiring pharmacological treatments, in particular those based on serotonin and noradrenaline reuptake inhibitors and/or selective serotonin reuptake inhibitors, given that they specifically impact inhibitory control;^23-26^ (vi) bipolar disorder, schizophrenia, history of drug or alcohol abuse. The Movement Disorder Society-Unified Parkinson’s Disease Rating Scale part 3 (MDS-UPDRS-III)^27^ was used to rate Parkinson’s disease motor symptoms in ON and OFF conditions. Levodopa equivalent daily dose (LEDD) was calculated according to the standard conversion table and separately for DA.^28^

We excluded 10 patients as they could not complete the test. The remaining patients (*n* = 60) were divided into three groups (*n* = 20 each) based on their H&Y stage assessed in ON condition: (i) H&Y 1–1.5 (from now on labelled as H&Y1), (ii) H&Y 2 (H&Y2), and (iii) H&Y 2.5–3 (H&Y3). To collect normative values of inhibitory control, we also tested 30 healthy controls (HCs). Average demographic, clinical characteristics, are reported in Table 1, Supplementary material, Paragraph 2, and Supplementary Table 2. Related statistics are reported in Supplementary Material, Paragraph 3, Supplementary Tables 3 and 4.

**Table 1:** Demographic and clinical features of Parkinson’s disease patients

	*n*	Age (years)	Sex (F/M)	Handedness (laterality score)	Education (years)	MMSE	Years since diagnosis	H&Y (ON)	Onset Side (R/L)	LEDD tot (mg)	LEDD DA (mg)	MDS-UPDRS-III (ON)	MDS-UPDRS-III (OFF)
H&Y1	20	61.5 (±6.8)	5/15	81.8 (±34.0)	11.5 (±3.6)	28.7 (±1.6)	3.6 (±1.9)	1.1 (±0.2)	9/11	343.9 (±175.1)	113.9 (±82.9)	9.9 (±5.1)	15.8 (±7.4)
H&Y2	20	61.5 (±9.1)	6/14	92.7 (±11.1)	11.5 (±3.7)	26.9 (±2.1)	5.2 (±2.8)	2	14/6	395.5 (±194.9)	66.1 (±83)	18.1 (±6.7)	29.7 (±8.2)
H&Y3	20	69.8 (±8.5)	8/12	95.5 (±8.9)	9.6 (±4.2)	27.3 (±2.2)	8.9 (±4.4)	2.8 (±0.2)	14/5^a^	685.8 (±286.5)	65.3 (±66.4)	22.6 (±9.5)	36.7 (±11.3)
HC	30	60.1 (±8.4)	18/12	89.7 (±13.0)	12.5 (±4.7)								

Patient groups are subdivided according to H&Y stages and shown with HCs as reference. For each group, mean age, sex, handedness,^19^ and years of education are shown. Furthermore, for each patient group, the mean of MMSE score, years since diagnosis, H&Y stages (indicating the stage of Parkinson’s disease disease, assessed with medication), the onset of Parkinson’s disease symptoms, total LEDD, LEDD DA, the total score of the MDS-UPDRS-III under pharmacological treatment (MDS-UPDRS-III, ON), and total UPDRS III score after an overnight wash-out of at least 12 h (MDS-UPDRS-III, OFF) are reported. ^a^One patient had a bilateral onset.

Briefly, the control group differed only from the H&Y3 group, as HCs were younger and had a longer education than H&Y3 patients. As expected, the clinical characteristics of patients differed as the H&Y1 patients (i) took the lowest dose of total LEDD, (ii) took the highest amount DA–LEDD and (iii) had the earliest onset of the disease. The severity of motor symptoms was evaluated in both ON and OFF states. As expected, motor symptoms worsened in the OFF condition overall. H&Y1 patients always had the lowest scores, both in ON and in OFF conditions, with respect to H&Y2 and H&Y3 patients. However, the MDS-UPDRS-III of H&Y2 and H&Y3 patients was not statistically different, although it was nominally higher in the latter than in the former. Finally, the DT differed across H&Y stages. Specifically, 11 patients took only DA and/or inhibitors of dopamine catabolism (H&Y1 = 9 DA; H&Y1 = 1 MAO B, H&Y2 = 1, DA). Twenty patients took only L-dopa (H&Y1 = 4; H&Y2 = 8; H&Y3 = 8). Twenty-nine patients took L-dopa and DA (H&Y1 = 6; H&Y2 = 11; H&Y3 = 12). Finally, the side-of-onset frequencies did not differ between patient groups.

All participants were naïve about the purpose of the study. The computed patient sample size was 60. The sample size of patients was established using a power analysis.^29^ Group means and standard deviations for the SSRT in the ON treatment condition were taken from previous studies.^3,4^ In line with Manza *et al*.,^30^ we expected that H&Y1 patients in OFF treatment increase the SSRT by 13% with respect to the ON condition. By contrast, we did not expect any significant differences between ON and OFF treatments^31^ in H&Y3 patients. We used a univariate approach to repeated measures with Geisser-Greenhouse correction and a target power of 0.8 (alpha = 0.05).

All participants provided written informed consent. The study was conducted following the ethical guidelines set forth by the Declaration of Helsinki and approved by the Institutional Ethics Committee of IRCCS Neuromed Hospital, Italy (NCT03665493).

### Behavioural tasks and procedure

We gave participants two tasks: (i) a reaction-time task (go-only task) and (ii) the reaching version of the SST, in a counterbalanced fashion. The behavioural tasks have been described in detail previously.^32^ Briefly, participants were seated in a dimly lit and silent room in front of a PC monitor coupled with a touchscreen (MicroTouch; sampling rate 200 Hz 3M Touch Systems Inc.) for touch-position monitoring.

**Figure 1:**
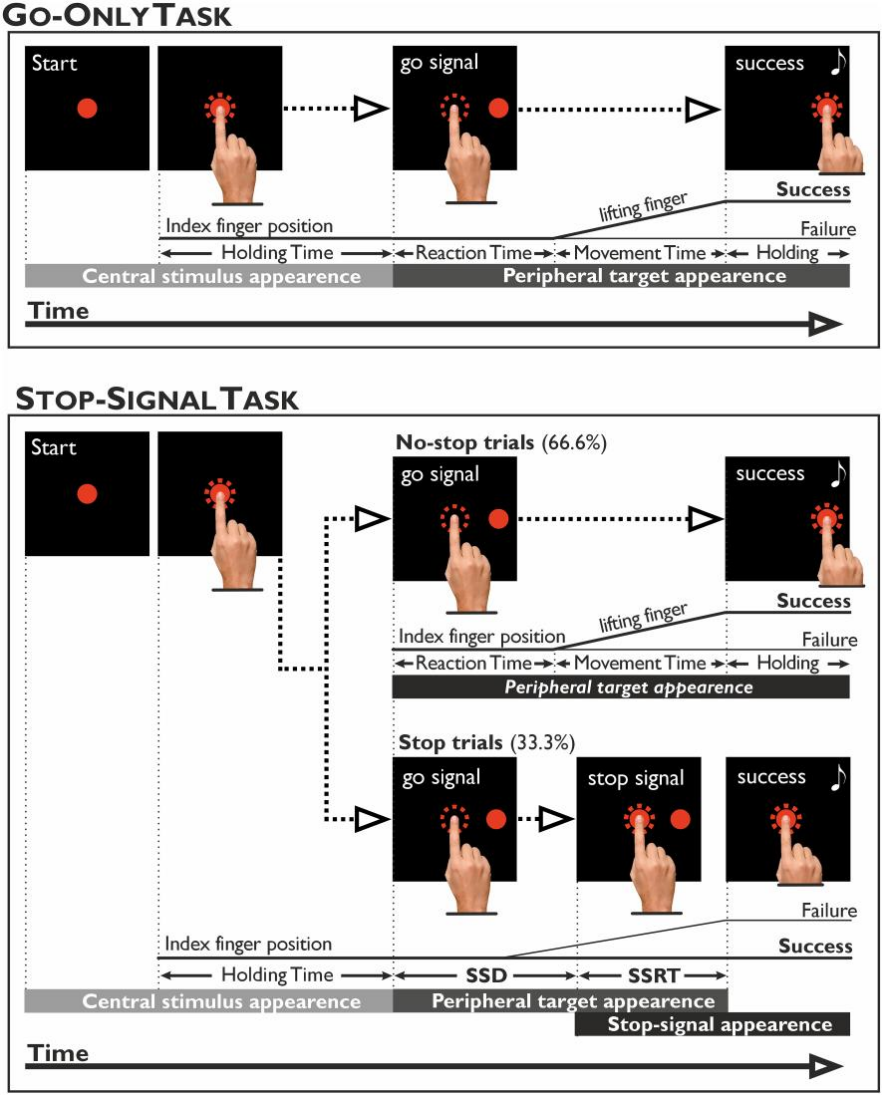
**Schematic depiction of the go-only task and the SST.** Go-Only task. Visual stimuli (targets) were displayed against a black background and consisted of red circles (4 dva). First, participants had to reach and hold (for 500–800 ms) the central target with the right index finger. Then, the central circle disappeared, and at 18.6 dva to the right, a peripheral target appeared (go-signal). Participants had to reach and hold it for 300–400 ms. SST. This task consisted of a pseudo-random intermix of no-stop trials (identical to the go-only task’s trials and consisted of 66.6% of the total trials) and stop trials (33.3%). In the stop trials, after the presentation of the go-signal, the central stimulus reappeared (stop signal) at a variable delay (SSD). Participants were instructed to inhibit the ongoing movement and to keep the index finger on the stop-signal for 300–400 ms. Correct trials were signalled by auditory feedback (represented by a white musical note). The dotted circle represented the size of the tolerance window for the touches (5 dva of diameter) and was invisible to the participants. The acquisition of behavioural responses and stimuli presentation was controlled by Cortex, a non-commercial software package developed at NIH (https://www.nimh.nih.gov/research/research-conducted-at-nimh/research-areas/clinics-and-labs/ln/shn/software-projects.shtml).

In the go-only task, participants were instructed to perform always a reaching movement (Fig. 1). Trials began with the appearance of a central red circle (luminance, 2.434 cd/m^2^; diameter: 4 degrees of visual angle, dva) against a dark background of uniform luminance (<0.01 cd/m^2^). Participants had to touch it with their index fingers. After a variable holding time (500–800 ms), the stimulus vanished, and another stimulus appeared 18.6 dva to the right on the horizontal plane (go-signal). Participants were instructed to respond to the go-signal by reaching the peripheral stimulus quickly and holding it for 300–400 ms. Acoustic feedback signalled correct trials. The SST (Fig. 1) consisted of a pseudorandom intermix of two types of trials, the no-stop trials, which were identical to the go-only trials and occurred 66% of the times, and the stop trials, which occurred less frequently (33% of times). In the stop trials, the central red circle reappeared (stop-signal) at a variable delay (stop-signal delay, SSD) after the go-signal presentation. In such instances, participants had to suppress the ongoing movement towards the peripheral target and hold their index finger on the central stimulus without lifting it for 300–400 ms. Successful trials were signalled by acoustic feedback. The SSD length started from 119.7 ms (nine refresh rates), and it was changed according to the participant’s performance via a staircase procedure.^33^ When participants correctly withheld the movement, the SSD was increased by 39.9 ms (three refresh rates), making stopping more difficult. Vice versa, when participants failed to inhibit, the SSD was decreased by the same amount of time, making stopping easier. This procedure aimed to keep the success rate of stop-signal trials around 50%. Importantly, as during the SST, participants tend to postpone their response to facilitate inhibition automatically,^34,35^ we inform them that the probability of successful stopping was set by an algorithm irrespective of their strategy. We also remarked that stop and no-stop trials were equally important and that they should not focus only on one type of trial. Furthermore, we set an reaction time (RT)-limit for no-stop trials to discourage the waiting strategy. Whenever the RTs exceeded 800 ms, no-stop trials were considered errors. However, to avoid cutting the RT distribution’s right tail,^35^ we gave participants an additional time of 100 ms for releasing the central stimulus. Thus, for trials not exceeding 900 ms, RTs were recorded and kept for the final analysis (overtime-reaching trials). Trials with RTs above 900 ms were aborted straight away.

The go-only task consisted of one block of 80–100 trials and the SST of four blocks of 108–120 trials each (432–480 trials overall). If requested, short breaks were interposed between the execution of blocks or tasks. The differences in the number of trials delivered were because we asked participants whether they were willing to do the long or the relatively shorter version at the beginning of the test. This was a psychological trick to encourage participants to perform our demanding tasks, especially for more advanced patients. Some patients did not complete all four blocks of the SST as they gave up earlier. In particular, one patient of the H&Y1 group and another of the H&Y2 group completed three blocks in ON and OFF medications. One patient of H&Y3 performed two blocks in ON and OFF medications. In addition, in the OFF state, three patients of the H&Y3 completed three blocks. Notably, in all cases, the number of stop trials was always above the minimum of 50 needed to compute a reliable estimate of inhibitory control behavioural parameters indicated by the most recent consensus guide.^9^

HCs performed the tasks in one session. Parkinson’s disease patients were tested in two separate and counterbalanced sessions, once in ON and another in OFF medication. These sessions were usually 1 week apart (5.9 ± 8.9 days). All patients, but the left-handed, performed the tasks with their dominant hand. The left-handed patient used the right hand, as it has been shown that the arm but not handedness impact inhibitory control proficiency.^36^

### Statistical analyses

To characterize participants’ behavioural performance, we analysed the following parameters: (i) the RTs, i.e. the time interval between go-signal appearance and movement onset; (ii) the movement times (MTs), i.e. the time interval between movement onset and the instant the peripheral target was touched; and (iii) the SSRT, computed using the intregration method as this provides the most reliable estimate^9^ (Supplementary material, Paragraph 4, Supplementary Fig. 2).

ANOVA were employed to assess changes in SSRTs, RTs and MTs. The proficiency of proactive inhibition was assessed by exploiting the phenomenon of the context effect.^37^ This refers to the fact that participants adaptively change their behavioural strategy when executing a reaching movement in the context of the go-only task or the SST. Although the fact that the required movement is the same when performing a no-stop trial, participants unconsciously increase the RTs, with respect to when executing a go-only trial, because of the possible appearance of a stop signal. By doing so, during no-stop trials, participants will likely have more time to compute the position of the peripheral target and hence decrease the MTs with respect to go-only trials.^37^ To evaluate the context effect, we employed three methods assessing differences in RTs and MTs, i.e. using ANOVAs on the mean values of RTs and MTs, and comparing the cumulative distributions of RTs and MTs by one-tailed two-sample Kolmogorov–Smirnov tests, both, at individual and population levels.

The assumption of normality was assessed using the Shapiro–Wilk test. Homoscedasticity was assessed by Levene’s test. Bonferroni corrections were applied to all multiple comparisons. The effect size was quantified by the partial eta-squared $\left(\eta_{p}^{2}\right)$ for the ANOVA and Cohen’s *d* for the *t*-test. We assessed the strength of null hypotheses by computing Bayes factors (BF_10_, *r*-scale = 0.707).^38^ BF_10_ values <0.33 and <0.1 provide moderate and robust support for the null hypothesis. Conversely, BF_10_ values >3 and >10 constitute moderate and strong support for the alternative hypothesis. Finally, BF_10_ Values between 0.33 and 3 are inconsistent for any hypothesis. However, it should be stressed that Bayes factors represent continuous evidence. Therefore, a BF_10_ of 0.58 indicates only slightly less moderate evidence than a Bayes factor 0.33. To evaluate differences in frequencies, we employed the *χ*^2^-test of homogeneity. Finally, we computed Spearman’s rank correlation test to assess correlations. Statistical analyses were conducted using R, version 4.0.0.^39^

## Results

We will report only significant results in the main text, while the complete set is provided in Supplementary Table 7.

### Data preprocessing

Trials with RTs shorter/longer than the mean ±3 SDs were discarded. We excluded 0.9 and 1.1% of HCs’ and patients’ no-stop trials in the SST, respectively. We excluded 3.5 and 4.9% of HCs’ and patients’ trials in the go-only task, respectively. In addition, overtime-reaching trials accounted for 9.2% of the total no-stop trials in HCs and 11.2% in patients. Data from overtime-reaching were included in the analyses (see Materials and methods section for more details).

### Race model assumptions checking

To assess whether collected data would provide a reliable estimate of the SSRT, we ensured whether (i) the assumption of the race model about the stochastic independence between the go-process (i.e. the process started by the go-signal eliciting movement initiation) and the stop-process (i.e. the process started by the stop-signal and eliciting movement inhibition), was fulfilled;^9,13^ (ii) the staircase algorithm’s performance worked as expected. Both conditions were satisfied (Supplementary material, Paragraph 5, Supplementary Table 5); thus, the SSRT estimates are accurate. Table 2 reports all relevant behavioural parameters.

**Table 2:** Summary of behavioural parameters for patients and HCs during the SST and the go-only task

Group	H&Y1		H&Y2		H&Y3		HC
treatment	OFF	ON	OFF	ON	OFF	ON	
SSRT	227.0 ± 34.1	252.3 ± 37.7	237.7 ± 29.4	254.6 ± 42.7	277.2 ± 39.0	267.7 ± 42.6	221.9 ± 25.7
P (failure)	0.49 ± 0.03	0.50 ± 0.04	0.50 ± 0.03	0.53 ± 0.05	0.54 ± 0.07	0.57 ± 0.10	0.51 ± 0.05
Mean SSD	376.3 ± 122.5	339.1 ± 123.3	277.2 ± 87.8	259.7 ± 128.0	240.1 ± 139.6	227.0 ± 135.0	255.0 ± 100.1
RT no-stop trials	614.4 ± 98.9	592.3 ± 105.2	531.6 ± 72.2	517.3 ± 127.0	513.6 ± 127.8	493.0 ± 136.4	492.3 ± 99.3
MT no-stop trials	473.8 ± 163	468.9 ± 128.8	664.2 ± 202.6	625.1 ± 219.7	634.9 ± 175.8	636.1 ± 220.7	443.8 ± 129.9
RT stop-failure trials	497.3 ± 93.2	476.6 ± 90.5	410.8 ± 54.5	395.1 ± 92.7	399.5 ± 100.9	388.6 ± 103.1	404.1 ± 82.9
RT go-only trials	267.8 ± 52.0	274.9 ± 58.3	294.1 ± 59.7	276.0 ± 53.8	279.3 ± 61.4	293.7 ± 65.9	306.5 ± 85.7
MT go-only trials	539.8 ± 216.4	539.5 ± 169.9	699.6 ± 207.6	634.7 ± 230.4	646.2 ± 141.4	659.7 ± 219.3	509.7 ± 152.2
Accuracy no-stop trials	0.88 ± 0.07	0.87 ± 0.13	0.87 ± 0.12	0.86 ± 0.11	0.84 ± 0.14	0.82 ± 0.12	0.89 ± 0.09
Accuracy go-only trials	0.90 ± 0.10	0.89 ± 0.1	0.90 ± 0.09	0.87 ± 0.17	0.86 ± 0.12	0.81 ± 0.17	0.91 ± 0.08

Mean behavioural values (±SD) for patients are reported for both, ON and OFF treatment conditions. Task accuracy is defined as the ratio between correct go-trials and the overall number of go-trials, i.e. sum of correct trials plus trials where participants missed the target or remained still on the central stimulus for more than 2 s or did not hold the central stimulus/target for the requested amount of time. Patients with H&Y stage 1–1.5 (H&Y1), 2 (H&Y3) and 2.5–3 (H&Y3). Abbreviations: HC, healthy controls; SSD, stop-signal delay; SSRT, stop-signal reaction time; RT, reaction time; MT, movement time; P (failure), probability of failing to perform a stop trial.

### Impact of DT on reactive inhibition

**Figure 2:**
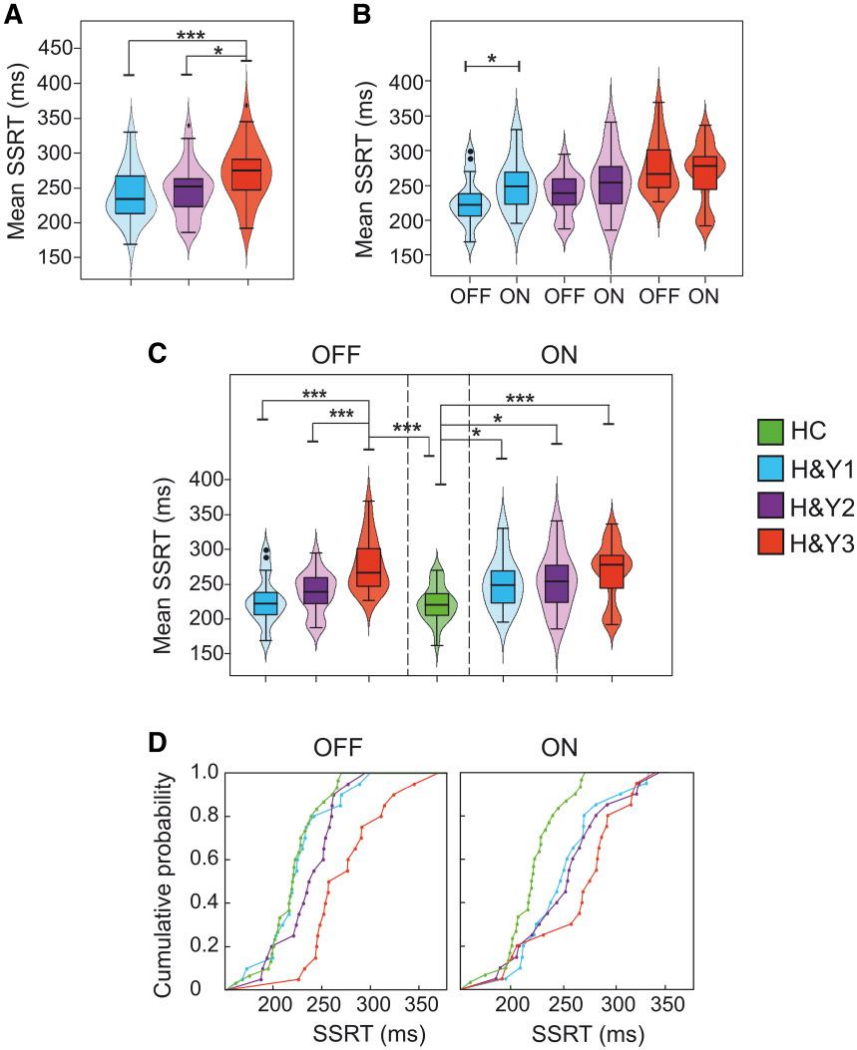
**Reactive inhibition in Parkinson’s’ patients groups (H&Y1, H&Y2, H&Y3, each *n* = 20) and HCs (*n* = 30).** Plots emphasize all the significant effects obtained in the statistical analyses. (**A**) Comparisons of SSRTs between patients at different H&Y stages after an overnight wash-out of at least 12 h (OFF); analysis: two-way mixed-design ANOVA (Group; Treatment), the main effect of the Group. (**B**) Comparisons of SSRTs between patients at different H&Y stages on OFF treatment and DT (ON); analysis: two-way mixed-design ANOVA (Group; Treatment), interaction effect Group × Treatment. (**C**) Comparisons of SSRTs between patients at different H&Y stages on OFF (left panel), ON treatment (right panel), and HC; analyses: two one-way between ANOVAs (Group), one comparing HC and Parkinson’s disease patients at different H&Y stages on OFF and ON treatment, respectively. (**D**) SSRT cumulative distributions of each patient’s group and HC. Left panel, patients on OFF treatment. Right panel, patients on DT (ON); analysis: two-sample Kolmogorov–Smirnov test. The violin plots depict kernel probability density, i.e. the areas’ width represents the data’s relative frequency. Box plots are shown inside the violin plots. The lower box’s boundary indicates the first quartile, the median is marked with a black line, and the upper box’s boundary indicates the third quartile. Whiskers indicate values 1.5 times the interquartile range below the first quartile and above the third quartile. Outliers are shown as black dots. *P < 0.05, ***P < 0.001.

We evaluated changes in the SSRT in Parkinson’s disease patients via a two-way mixed-design ANOVA [between-participants factor: Group (H&Y1, H&Y2, and H&Y3); within-participant factor: Treatment (ON and OFF)]. We found a main effect of the Group (Table 3, Fig. 2A) because H&Y3 patients had longer SSRTs (272.4 ± 40.6 ms) with respect to H&Y1 (239.7 ± 37.7 ms) and H&Y2 patients (246.2 ± 37.2 ms). We also had a significant Group × Treatment interaction since H&Y1 patients exhibited a shorter average SSRT in the OFF than ON condition. Instead, H&Y2 and H&Y3 patients did not differ (Tables 2 and 3, Fig. 2B). To compare the reactive inhibition of patients in OFF and ON conditions with HCs, we ran two one-way ANOVAs on the SSRTs [between-participants factor: Group (HC, H&Y1, H&Y2, and H&Y3)]. In both ANOVAs, we found a main effect (Fig. 2C). Pairwise comparisons in the OFF condition showed that HCs, H&Y1, and H&Y2 patients had a shorter SSRT than H&Y3 patients. Differently, HCs showed a shorter average SSRT than patients in the ON condition. Instead, no differences between Parkinson’s disease patients in the ON condition occurred.

**Table 3:** Statistical analysis results of SSRTs in Parkinson’s groups and HCs

Two-way ANOVA on SSRT between-participant factors: Group (H&Y1, H&Y2, and H&Y3); within-participant factors: Treatment (ON and OFF)							
		Value of parameters	*P-*values	*M*_diff_	95% CI	Effect size	BF_10_
Main effect	Group	*F*(2,57) = 6.13	**0.004**			$\eta_{p}^{2}$ = 0.18	10.38
*Post hoc* tests	H&Y1 versus H&Y3	*t*(57) = −3.31	**0.005**	−32.8	(−50.2, −15.3)	*d* = 0.84	74.14
	H&Y2 versus H&Y3	*t*(57) = −2.65	**0.031**	−26.3	(−43.6, −8.9)	*d* = 0.67	10.65
Interaction	Group × Treatment	*F*(2,57) = 3.61	**0.033**			$\eta_{p}^{2}$ = 0.11	1.99
*Post hoc* tests	H&Y1 OFF versus H&Y1 ON	*t*(57) = −2.65	**0.031**	−25.3	(−41.3, −9.2)	*d* = 0.74	11.67

One-way ANOVA on SSRT in OFF condition between-participant factor: Group (HC, H&Y1, H&Y2, and H&Y3)							
Main effect	Group	*F*(3,86) = 13.49	**<0.001**			$\eta_{p}^{2}$ = 0.32	>100
*Post hoc* tests	HC versus H&Y3	*t*(86) = −6.03	**<0.001**	−55.3	(−75.5, −35.0)	*d* = 1.75	>100
	H&Y1 versus H&Y3	*t*(86) = −4.99	**<0.001**	−50.1	(−73.6, −26.6)	*d* = 1.37	>100
	H&Y2 versus H&Y3	*t*(86) = −3.93	**0.001**	−39.5	(−61.6, −17.3)	*d* = 1.14	34.67

One-way ANOVA on SSRT in ON condition between-participant factor: Group (HC, H&Y1, H&Y2, and H&Y3)							
Main effect	Group	*F*(3,86) = 7.24	**<0.001**			$\eta_{p}^{2}$ = 0.20	>100
*Post hoc* tests	HC versus H&Y1	*t*(86) = −2.88	**0.030**	−30.4	(−50.1, −10.7)	*d* = 0.98	23.78
	HC versus H&Y2	*t*(86) = −3.1	**0.016**	−32.8	(−54.6, −11.0)	*d* = 0.98	23.41
	HC versus H&Y3	*t*(86) = −4.33	**<0.001**	−45.8	(−67.6, −24.1)	*d* = 1.37	>100

Only statistically significant results are reported (in bold). *Post hoc* tests (pairwise comparisons) had an adjusted alpha level corrected according to Bonferroni. Bayes factors indicate the ratio between the null and alternative hypothesis (BF_10_). Effect sizes are reported as partial eta squared $\left(\eta_{p}^{2}\right)$ and Cohen’s *d*. Patients’ assessment was performed under pharmacological treatment (ON) and after an overnight wash-out of at least 12 h (OFF). Abbreviations: SSRT, stop signal reaction time; CI, confidence interval; H&Y, Hoehn and Yahr groups 1–1.5, 2, and 2.5–3 (H&Y1, H&Y2, and H&Y3, respectively); HCs, healthy controls.

The overall pattern can be better appreciated by looking at the cumulative distributions of the SSRTs (Fig. 2D). In the OFF state, the cumulative distribution of HCs was not different from that of H&Y1 (two-sample Kolmogorov–Smirnov test, *D* = 0.17, *P* = 0.86) and H&Y2 (*D* = 0.35, *P* = 0.081) patients, but it differs from that of H&Y3 patients (*D* = 0.7, *P* < 0.0001). In the ON state, the cumulative distribution of HCs becomes different with respect to those of all patient groups (H&Y1, *D* = 0.43, *P* = 0.015; H&Y2, *D* = 0.45, *P* = 0.01; H&Y3, *D* = 0.61, *P* < 0.0001). At the same time, when comparing the cumulative distributions in ON and OFF states within each patient’s group, only H&Y1 differed (two-sample Kolmogorov–Smirnov test, *D* = 0.45, *P* = 0.023). In contrast, H&Y2 (*D* = 0.3, *P* = 0.27) and H&Y3 (*D* = 0.25, *P* = 0.49) patients did not show differences. Altogether, these results suggest that the DT severely impairs reactive inhibitory control in H&Y1 patients.

Finally, as patients received different types of dopaminergic medications, we wanted to assess whether reactive inhibition was affected by the pharmacological treatment, irrespective of the disease stage. Thus, we check for changes in the SSRT using a two-way mixed-design ANOVA [between-participants factor: Therapy (only L-dopa, only DA, and L-dopa, DA & L-dopa, DA); within-participant factor: Treatment (ON and OFF)]. We found a main effect of Treatment because the SSRT was longer in ON than in OFF condition. There was also a main effect of Therapy; however, this effect did not survive *post hoc* comparisons (Supplementary Table 6). Relevantly, the interaction Treatment × Therapy was not significant, meaning that the effect of the DT was not linked to the type of medication received by patients.

### Impact of DT on proactive inhibition

To evaluate proactive inhibition, we measured the context effect^37^ using three approaches, two at the population level and one at the single individual level.

#### Context effect at the population level: comparison of the means of RTs and MTs

**Figure 3:**
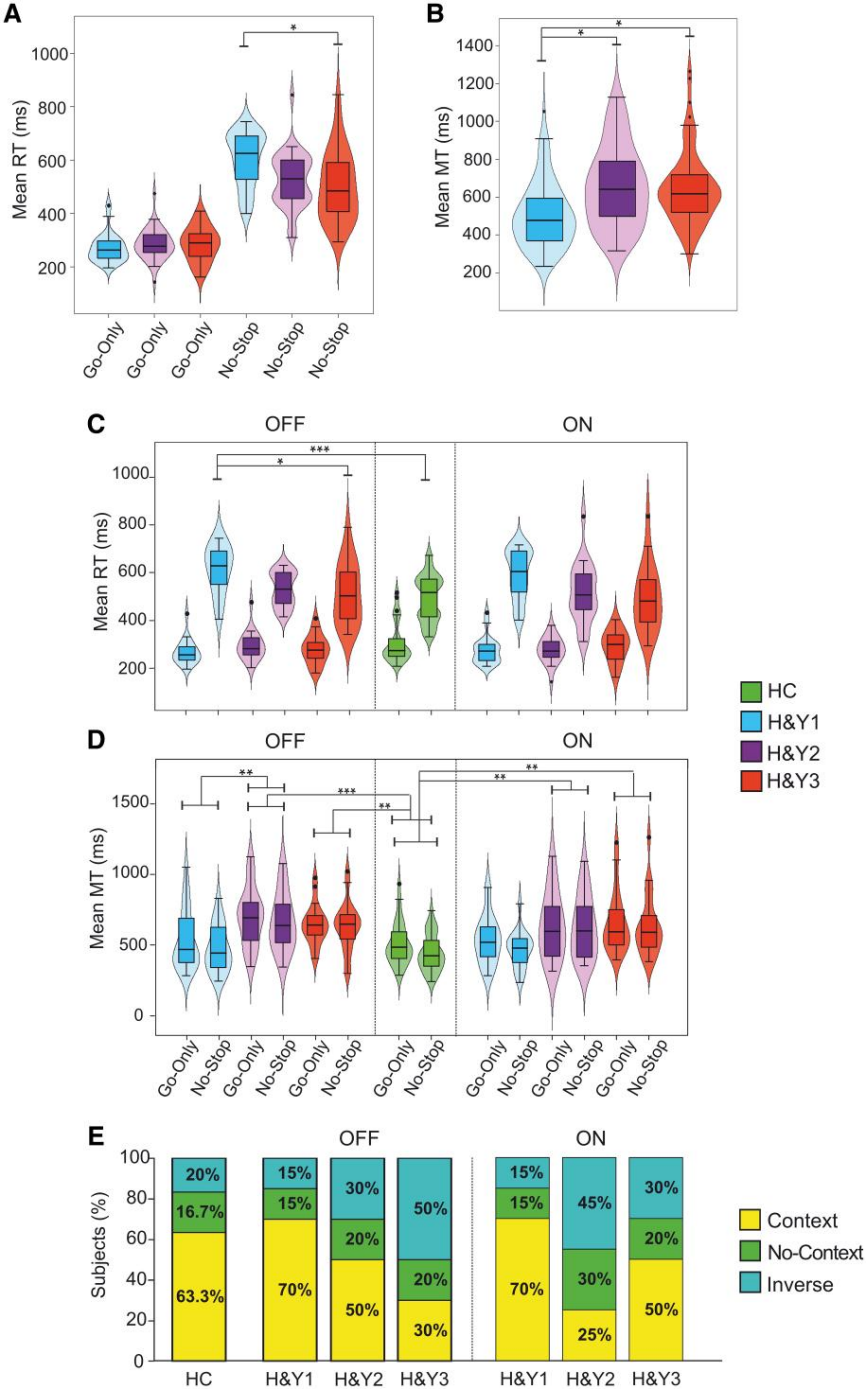
**Proactive inhibition in Parkinson’s’ patients groups (H&Y1, H&Y2, H&Y3, each *n* = 20) and HCs (*n* = 30).** Plots emphasize all the significant effects obtained in the statistical analyses. (**A**) Comparisons between RTs of patient groups in no-stop and go-only trials; analysis: three-way mixed-design ANOVA (Group, Treatment, Trial type), interaction effect Group × Trial type. (**B**) Comparisons between MTs of patient groups; analysis: three-way mixed-design ANOVA (Group, Treatment, Trial type), main effect of Group. (**C**) Comparisons of RTs between patients at different H&Y stages after an overnight wash-out of at least 12 h (OFF, left panel) and on DT (ON, right panel) and HC; analyses: two three-way mixed-design ANOVA (Group, Treatment, Trial type), comparing HC and Parkinson’s disease patients at different H&Y stages on OFF and ON treatment, respectively; interaction effect Group × Treatment × Trial type. (**D**) Comparisons of MTs between patients at different H&Y stages on OFF (left panel) and ON treatment (right panel) and HC; analysis: two three-way mixed-design ANOVA (Group, Treatment, Trial type), comparing HC and Parkinson’s disease patients at different H&Y stages on OFF and ON treatment, respectively; interaction effect Group × Treatment × Trial type. (**E**) Percentage of participants showing a context effect, a no-context effect, or an inverse context effect. See text for more details. All conventions are as in Fig. 2. *P < 0.05, **P < 0.01, and ***P < 0.001.

The first population analysis compared the means of RTs and MTs of no-stop and go-only trials with three mixed-design ANOVAs. The first one included only patients and was a three-way ANOVA [between-participants factors: Group (H&Y1, H&Y2, and H&Y3); within-participant factors: Treatment (ON and OFF), Trial type (RT no-stop trials, RT go-only trials)]. As shown in Table 4, we found a main effect of Trial type because, as expected, the RTs of no-stop trials were significantly longer than those of go-only trials (Fig. 3A and C). We also found a significant interaction Group × Trial type, indicating that the RTs of no-stop trials of H&Y1 patients were significantly slower (603.4 ± 101.4 ms) than those of H&Y3 patients (503.3 ± 130.9 ms, Fig. 3A). Nominally, H&Y1 patients also had longer RTs than H&Y2 patients. In addition, the RTs of no-stop trials were longer than those of go-only trials in each patient group.

**Table 4:** Results of the statistical analysis of RTs and MTs across Parkinson’s disease patient groups and controls

Three-way ANOVA on RT between-participants factors: Group (H&Y1, H&Y2, and H&Y3); within-participant factors: Treatment (ON and OFF); Trial type (no-stop and go-only)							
		Value of parameters	*P-*values	*M*_diff_	95% CI	Effect size	BF_10_
Main effect	Trial type	*F*(1,57) = 378.59	**<0.001**	262.8	(239.8, 285.7)	$\eta_{p}^{2}$ = 0.87	>100
Interaction	Group × Trial type	*F*(2,57) = 6.81	**0.002**			$\eta_{p}^{2}$ = 0.19	>100
*Post hoc* test	No-stop H&Y1 versus H&Y3	*t*(57) = 3.12	**0.025**	100.0	(47.9, 152.2)	*d* = 0.85	94.76
	H&Y1 no-stop versus go-only	*t*(57) = 14.20	**<0.001**	332.0	(297.7, 366.3)	*d* = 3.03	>100
	H&Y2 no-stop versus go-only	*t*(57) = 10.23	**<0.001**	239.4	(200.4, 278.3)	*d* = 1.97	>100
	H&Y3 no-stop versus go-only	*t*(57) = 9.27	**<0.001**	216.8	(177.5, 256.2)	*d* = 1.76	>100

Two-way ANOVA on RT in OFF condition between-participant factors: Group (HC, H&Y1, H&Y2, and H&Y3) within-participant factors: Trial type (no-stop and go-only)							
Main effect	Trial type	*F*(1,86) = 454.59	**<0.001**	251.1	(218, 269.6)	$\eta_{p}^{2}$ = 0.841	>100
Interaction	Group × Trial type	*F*(3,86) = 8.68	**<0.001**			$\eta_{p}^{2}$ = 0.232	>100
*Post hoc* tests	No-stop HC versus H&Y1	*t*(86) = −4.18	**0.001**	−122.2	(−179.9, −64.5)	*d* = 1.23	>100
	No-stop H&Y1 versus H&Y3	*t*(86) = 3.15	**0.036**	100.8	(27.5, 174.1)	*d* = 0.88	5.79
	HC no-stop versus go-only	*t*(86) = −9.25	**<0.001**	185.8	(146.4, 225.2)	*d* = 1.76	>100
	H&Y1 no-stop versus go-only	*t*(86) = −14.09	**<0.001**	346.6	(298.2, 395.0)	*d* = 3.35	>100
	H&Y2 no-stop versus go-only	*t*(86) = −9.66	**<0.001**	237.5	(186.0, 289.0)	*d* = 2.16	>100
	H&Y3 no-stop versus go-only	*t*(86) = −9.53	**<0.001**	234.3	(177.0, 291.6)	*d* = 1.91	>100

Two-way ANOVA on RT in ON condition between-participant factors: Group (HC, H&Y1, H&Y2, and H&Y3) within-participant factors: Trial type (no-stop and go-only)							
Main effect	Trial type	*F*(1,86) = 347.79	**<0.001**	230.4	(203.8, 257)	$\eta_{p}^{2}$ = 0.802	>100
Interaction	Group × Trial type	*F*(3,86) = 5.55	**0.002**			$\eta_{p}^{2}$ = 0.162	31.66
*Post hoc* tests	HC no-stop versus go-only	*t*(86) = −8.61	**<0.001**	185.8	(146.4, 225.2)	*d* = 1.76	>100
	H&Y1 no-stop versus go-only	*t*(86) = −12.01	**<0.001**	317.4	(265.1, 369.8)	*d* = 2.84	>100
	H&Y2 no-stop versus go-only	*t*(86) = −9.13	**<0.001**	241.3	(177.9, 304.7)	*d* = 1.78	>100
	H&Y3 no-stop versus go-only	*t*(86) = −7.54	**<0.001**	199.4	(141.2, 257.5)	*d* = 1.61	>100

Three-way ANOVA on MT between-participant factors: Group (H&Y1, H&Y2, and H&Y3); within-participant factors: Treatment (ON and OFF); Trial type (no-stop and go-only)							
Main effect	Group	*F*(2,57) = 5.32	**0.008**			$\eta_{p}^{2}$ = 0.16	6.68
*Post hoc* tests	H&Y1 versus H&Y2	*t*(57) = −2.93	**0.015**	−150.4	(−211.0, −89.8)	*d* = 0.78	>100
	H&Y1 versus H&Y3	*t*(57) = −2.70	**0.027**	−138.7	(−195.2, −82.2)	*d* = 0.77	>100
Main effect	Trial type	*F*(1,57) = 5.43	**0.023**	−36.1	(−61.7, −10.5)	$\eta_{p}^{2}$ = 0.09	1.57

Two-way ANOVA on MT in OFF condition between-participants factors: Group (HC, H&Y1, H&Y2, and H&Y3). within-subj. factors: Trial type (no-stop and go-only)							
Main effect	Group	*F*(3,86) = 8.99	**<0.001**			$\eta_{p}^{2}$ = 0.239	>100
*Post hoc* tests	HC versus H&Y2	*t*(86) = −4.45	**<0.001**	−205.1	(−279.3, −131)	*d* = 1.21	>100
	HC versus H&Y3	*t*(86) = −3.55	**0.004**	−163.8	(−225.7, −101.9)	*d* = 1.09	>100
	H&Y1 versus H&Y2	*t*(86) = −3.47	**0.005**	−175.1	(−263.2, −87.1)	*d* = 0.89	>100
Main effect	Trial type	*F*(1,86) = 10.25	**0.002**	−44.70	(−74.3, −19.8)	$\eta_{p}^{2}$ = 0.106	26.7

Two-way ANOVA on MT in ON condition between-participant factors: Group (HC, H&Y1, H&Y2, and H&Y3). within-subj. factors: Trial type (no-stop and go-only)							
Main effect	Group	*F*(3,86) = 5.86	**<0.001**			$\eta_{p}^{2}$ = 0.170	32.3
*Post hoc* tests	HC versus H&Y2	*t*(86) = −3.08	**0.016**	−153.1	(−232.6, −73.6)	*d* = 0.85	>100
	HC versus H&Y3	*t*(86) = −3.45	**0.005**	−171.1	(−249.3, −92.9)	*d* = 0.97	>100
Main effect	Trial type	*F*[1,86] = 9.58	**0.003**	−42.5	(−72.0, −18.1)	$\eta_{p}^{2}$ = 0.100	20.0

Only statistically significant results are reported (in bold, for complete statistics see Supplementary Table 7). All *post hoc* tests (pairwise comparisons) had an adjusted alpha level corrected according to Bonferroni. Bayes factors report the ratio between the null versus the alternative hypothesis (BF_10_), Effect sizes are reported as partial eta squared $\left(\eta_{p}^{2}\right)$ and Cohen’s *d*. Abbreviations: CI, confidence interval; H&Y groups 1–1.5, 2, and 2.5–3 (H&Y1, H&Y2, and H&Y3, respectively); HCs, healthy controls. Assessment performed under pharmacological treatment (ON), and after an overnight wash-out of at least 12 h (OFF).

The same analyses on MTs showed a significant main effect of Trial type and Group (Table 4, Fig. 3D). The former result was because, as expected, participants had longer MTs during go-only trials than no-stop trials. The effect of Group was due to H&Y1 patients moving significantly faster (471.3 ± 145 ms) than H&Y2 (644.6 ± 209.5 ms) and H&Y3 (635.5 ± 196.9 ms) patients in no-stop trials (Fig. 3B).

We compared average RTs and MTs of Parkinson’s disease patients in OFF and ON conditions with those of HCs via two two-way ANOVAs [between-participants factors: Group (HC, H&Y1, H&Y2, and H&Y3); within-participant factor: Trial type (RT/MT no-stop trials and RT/MT go-only trials)]. The comparison between the RTs of HCs and Parkinson’s disease patients in the OFF state revealed a significant main effect of Trial type and interaction Group × Trial type (Table 4). The former effect was because no-stop trials RTs were always longer than go-only trials RTs. The interaction indicated that no-stop trials RTs of H&Y1 patients were significantly longer (614.4 ± 98.9 ms) than HCs (492.3 ± 99.3) and H&Y3 patients (513.6 ± 127.8 ms, Fig. 3C). The same analysis on HCs and Parkinson’s disease patients in the ON state also revealed a significant main effect of Trial type and interaction Group × Trial type. Both effects were due to the no-stop trials RTs being always longer than go-only trials RTs in all groups (Table 4).

The two-way ANOVA analyses on MTs of HCs and patients in the OFF and ON conditions revealed two significant main effects of Trial type and Group (Fig. 3D). The former effect was because MTs in No-stop trials were always longer than those in go-only trials. The effect of Group was because HCs (443.8 ± 129.9 ms) were faster than H&Y2 and H&Y3 patients (OFF state: 664.2 ± 202.6 ms and 634.9 ± 175.8 ms; ON state: 625.1 ± 219.7 ms, and 636.1 ± 220.7 ms). In addition, in the OFF state, H&Y1 patients were faster (473.8 ± 163 ms) than H&Y2 patients (664.2 ± 202.6 ms).

These results indicate that although the context effect was overall present, H&Y1 patients tend to show a larger effect than all other patient groups, as they have longer RTs in no-stop trials and, correspondingly, shorter MTs than H&Y2 and H&Y3 patients. Therefore, H&Y1 patients optimize the waiting tendency to enhance the probability of inhibiting motor responses. However, when they decide to move, they are faster, probably because in the extra-waiting time, they are more capable of computing the position of the peripheral target.

To check whether the type of dopaminergic medication received by patients affects the RTs or the MTs irrespective of the disease stage, we run a three-way mixed-design ANOVA [between-participants factor: Therapy (L-dopa, DA & L-dopa, DA); within-participant factor: Treatment (ON and OFF)]. Regarding the RTs, we found only an effect of Trial type because RTs of no-stop trials were longer than those of go-only trials (Supplementary Table 6). Instead, we found a main effect of Therapy for the MTs since patients taking dopamine-agonists were faster than those taking only L-dopa. Crucially, we never found significant interactions between Therapy and Treatment, suggesting that the observed effects were not due to the assumption of a specific kind of pharmacological treatment. In other words, the medication type does not influence proactive inhibition.

#### Context effect at population level: population cumulative distributions

**Figure 4:**
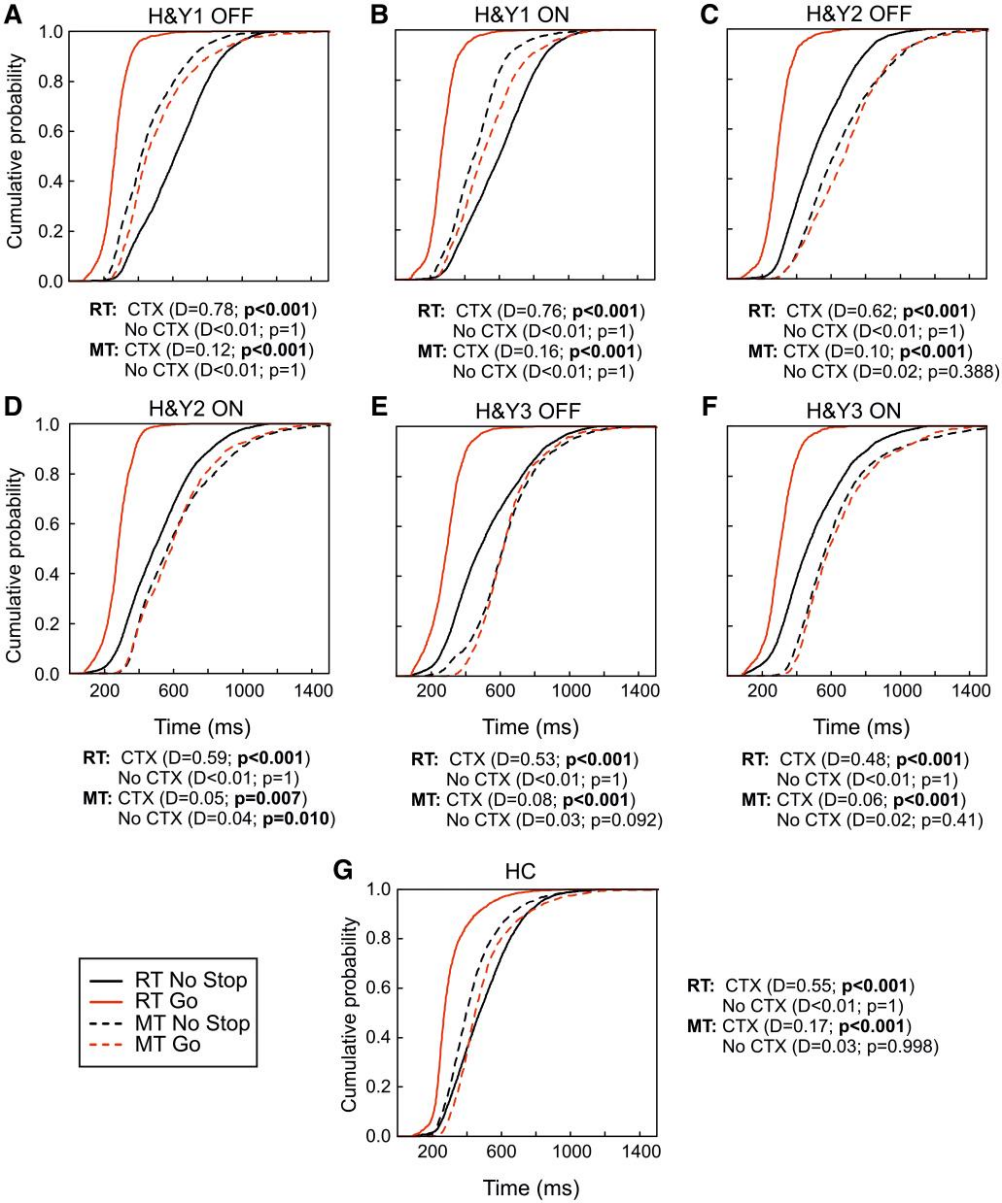
**Proactive inhibition: cumulative distributions of patient populations and HCs.** Cumulative distributions of no-stop (black lines) and go-only trials (red lines) (RTs, solid lines) and (MTs, dashed lines) of Parkinson’s’ patients subdivided according to the H&Y stage: 1–1.5 (H&Y1, *n* = 20), 2 (H&Y2, *n* = 20), and 2.5–3 (H&Y3, *n* = 20) and HC (*n* = 30). (**A**) H&Y1 patients without DT (OFF) and (**B**) with DT. (**C**) H&Y2 patients in OFF and (**D**) in ON condition. (**E**) H&Y3 in OFF and (**F**) in ON condition. (**G**) HC. Cumulative distributions were obtained by collapsing together those of single participants. *P*-values and Kolmogorov’s *D*-statistics of the one-sided two-sample Kolmogorov–Smirnov test are reported. CTX, context effect; No CTX, absence of context effect.

The second population approach was based on creating cumulative population distributions of RTs and MTs of go-only trials and no-stop trials by combining cumulative distributions of single participants (Fig. 4). We ran two one-tailed two-sample Kolmogorov–Smirnov tests to assess differences between cumulative distributions. In one comparison, the null hypothesis coincided with the predictions of the context effect, i.e. (i) the distribution of the go-only trials RTs was on the left of the distribution of no-stop trials RTs; (ii) the distribution of the go-only trials MTs was on the right of the distribution of no-stop trials MTs. In the other comparison, the null hypothesis was the opposite of what was predicted by the context effect. Such an approach is needed to reveal when an intersection between the two distributions occurs, indicating an absence of an effect.

In HCs (Fig. 4G), in H&Y1 and H&Y3 patients in ON and OFF states (Fig. 4A, B, E, and F), and in H&Y2 patients in OFF condition (Fig. 4C), the predictions of the context effect were always verified. By contrast, in H&Y2 patients in ON state (Fig. 4D), while the go-only trials RTs distribution was on the left of the no-stop trials RTs distribution, the no-stop and go-only trials MTs distributions were significant in both comparisons, i.e. the two distributions intersected and thus at the same time the go-only trials MTs distributions was on the right of the distribution of the no-stop trials and vice versa. We defined this instance as no context effect because there was no indication about which distribution is different, i.e. MTs of no-stop trials were not different from those of go-only trials. These results suggest that, although with different magnitudes, the context effect was present in all groups but in H&Y2 patients in the ON state. In these patients, the DT impairs proactive inhibition.

#### Context effect at individual level

The individual-level approach was based on the analyses of individual cumulative distributions of RTs and MTs of no-stop and go-only trials. For each participant, via a two-sample Kolmogorov–Smirnov test, we assessed whether a simultaneous increase in RTs and decrease in MTs in no-stop trials with respect to go-only trials occurred. Then, we computed the percentage of participants exhibiting a context effect, a no-context effect (i.e. MTs of no-stop trials were not different from those of go-only trials), or an inverse context effect (i.e. MTs of no-stop trials were longer than those of go-only trials; Fig. 3E).

We assessed differences in the context effect frequency between participant groups via a *χ*^2^ test of homogeneity (*n* = 90; Table 5). This analysis showed significant results both in OFF and in ON conditions. *Post hoc* tests on standard residuals in the OFF condition did not survive Bonferroni correction, whereas, in the ON condition, the H&Y2 group deviated from expected frequencies due to a smaller percentage of occurrences of the context effect.

**Table 5:** Chi-square test of homogeneity across all participant groups

Chi-square tests of homogeneity between group [H&Y1, H&Y2, H&Y3, and HC] and presence of context effect					
	Treatment	*Post hoc* or pairwise test	Value of parameters	*P-*values	BF_10_
			*χ* ^2^(3) = 7.88	**0.048**	1.53
*Post hoc* tests on standard residuals	OFF	H&Y1 OFF	stRes = 1.58	0.453	NA
	OFF	H&Y2 OFF	stRes = −0.45	1	NA
	OFF	H&Y3 OFF	stRes = −2.49	0.051	NA
		HC	stRes = 1.20	0.925	NA
			*χ* ^2^(3) = 9.98	**0.019**	4.45
*Post hoc* tests on standard residuals	ON	H&Y1 ON	stRes = 1.69	0.361	NA
	ON	H&Y2 ON	stRes = −2.88	**0.016**	NA
	ON	H&Y3 ON	stRes = −0.34	1	NA
		HC	stRes = 1.34	0.715	NA

Chi-square tests of homogeneity between Parkinson’s disease patients at H&Y stage 1–1.5 (H&Y1), 2 (H&Y3), 2.5–3 (H&Y3), and HCs showing the ‘context effect’.^37^ Bayes factors showing the likelihood of the alternative versus the null hypothesis (BF_10_) were computed using the Bayesian analyses of contingency tables.^40^ It is not possible to calculate BF_10_ on standard residuals (stress). Statistically significant results are reported in bold. Abbreviation: NA, not applicable.

To compare patient groups with HCs, we ran a series of chi-square tests of independence (Table 6). HCs had a higher frequency of context effect than H&Y3 patients in the OFF condition and H&Y2 patients in the ON condition.

**Table 6:** Chi-square test of independence comparing patient’s groups and HCs

Chi-square tests of independence for HC (*n* = 30) versus PD patient groups (*n* = 60) and context effect (present and absent)					
Comparison	Treatment	*N*	Value of parameters	*P-*values	BF_10_
HC versus H&Y1	OFF	50	*χ* ^2^(1) = 0.23	0.629	0.36
HC versus H&Y2	OFF	50	*χ* ^2^(1) = 0.86	0.354	0.52
HC versus H&Y3	OFF	50	*χ* ^2^(1) = 5.23	**0.022**	4.68
HC versus H&Y1	ON	50	*χ* ^2^(1) = 0.23	0.629	0.36
HC versus H&Y2	ON	50	*χ* ^2^(1) = 6.92	**0.009**	11.24
HC versus H&Y3	ON	50	*χ* ^2^(1) = 0.86	0.354	0.52

Chi square tests of independence with *N−*1 correction,^41^ between Parkinson’s disease patients with H&Y stage 1–1.5 (H&Y1), 2 (H&Y3), and 2.5–3 (H&Y3), and HCs. Statistically significant results are reported in bold. Bayes factors showing the likelihood of the alternative versus the null hypothesis (BF_10_) were computed using the Bayesian analyses of contingency tables.^40^

Overall, these analyses indicate that DT selectively impairs proactive inhibition in H&Y2 patients.

#### The findings yielded by the three distinct approaches for evaluating proactive inhibition are in agreement with each other

The three different approaches for evaluating proactive inhibition produced comparable qualitative findings. First, H&Y1 patients always exhibit a proactive inhibitory control nominally better than HCs and all other patient groups in the ON and OFF conditions. Second, H&Y2 patients showed a marked impairment of proactive inhibition in the ON condition. Third, H&Y3 patients show better proactive inhibition in ON than in OFF conditions.

#### The context effect is not a speed-accuracy trade-off phenomenon

As it has been shown that faster responses tend to increase the number of errors, the so-called speed-accuracy trade-off phenomenon,^42^ we checked whether the accuracy of no-stop trials differed from that of go-only trials (Table 2). For patients, we used a three-way mixed-design ANOVA [between-participants factor: Group (H&Y1, H&Y2, and H&Y3); within-participant factors: Accuracy (no-stop trials and go-only trials) and Treatment (ON and OFF)]. For HCs, we used a paired samples Wilcoxon test. No significant effects were found either in the ANOVA or in the Wilcoxon test. Therefore, we concluded that there was no difference in the accuracy between no-stop and go-only trials.

## Discussion

For the first time, we assessed the effect of the DT on the two domains of motor inhibitory control, i.e. reactive and proactive inhibition, using a within-participant design, comparing patients’ performance on a simple reaction-time task and a SST both in ON and in OFF states in a counterbalanced fashion, and evaluating the impact of the pharmacological treatment on different stages of the disease. Our main findings are the following: (i) the influence of DT varies based on the disease stage because its administration selectively impairs inhibitory control in the earliest stages of the disease by weakening reactive inhibition in the H&Y1 stage and proactive inhibition in the H&Y2 stage, whereas it exerts some slightly beneficial effects on proactive inhibitory domain in H&Y3 patients; (ii) the effects of DT do not appear to depend on the specific type of dopaminergic drugs used (DA or L-dopa) or on the combination of such drugs; (iii) inhibitory proficiency can serve as a valuable marker of disease progression when patients are in the OFF therapy state because a progressive deterioration of both reactive and proactive components along the disease course occurs. Notably, on the one hand, all significant results are sustained by large effect sizes and by values of the BF_10_, providing strong support for the alternative hypothesis. On the other hand, all crucial, significant results are supported by values of the BF_10_, indicating a higher likelihood of the null hypothesis. Therefore, we can state our evidence is statistically and methodologically very solid.

### Changes of inhibitory proficiency during disease progression

Using a stage-dependent approach, we showed changes in reactive and proactive inhibition during Parkinson’s disease progression net of DT. In the OFF condition, we found that inhibitory control progressively deteriorates throughout the disease. At stage H&Y1, reactive inhibition is similar to HCs, while proactive inhibition is enhanced even with respect to HCs because patients could prolong their RTs more effectively than them. In H&Y2 patients, the SSRT is nominally longer than in HCs and H&Y1 patients (Table 2), even though not significantly different. Proactive inhibition also starts to deteriorate as the individual frequency of the context effect occurrences decreases (Fig. 3E), the RTs of no-stop trials become nominally faster, and the MTs shorter than those of H&Y1 patients. In other words, H&Y2 patients become more impulsive. At H&Y3, there is a marked deterioration of both reactive and proactive inhibitory control. The correlation between patients’ symptoms severity, measured on the MDS-UPDRS-III, with the behavioural parameters characterizing the SST (i.e. the SSRTs, the RTs, and MTs of no-stop signal trials) fully supports the observation of progressive impairment of inhibitory control along the course of the disease (Supplementary material, Paragraph 7, Supplementary Fig. 3).

These results partially agree with our previous study.^3^ As in the study by Di Caprio *et al*.,^3^ proactive inhibition was intact, however, we also found that the reactive domain was intact, while previously, we showed an impairment of reactive inhibition. This discrepancy is likely because in the study by Di Caprio *et al*.,^3^ ∼40% of patients were tested in the ON state, and the others were *de novo* patients, i.e. drug naïve. In sum, our findings suggest that the impairment in response inhibition is contingent on the disease stage. Thus, inhibitory proficiency, measured in the absence of DT, can serve as a valuable marker of disease progression, given that it consistently diminishes over the course of the illness.

### Effect of dopaminergic therapy on motor inhibition

In the earliest stages of Parkinson’s disease, DT significantly impairs inhibitory control, but it does not affect patients in the H&Y3 stage. Patients in the H&Y1 stage exhibit a notable decline in reactive inhibition proficiency in the ON state compared with that in the OFF state. However, proactive inhibition becomes even slightly more effective than in HCs. We propose that this enhancement partially compensates for the deficit in reactive inhibition. In the H&Y2 stage, DT has a minor, albeit non-significant, impact on prolonging the SSRT (Table 2). However, it significantly impairs proactive inhibition, paradoxically becoming even less efficient than in the H&Y3 stage. In contrast, among H&Y3 patients, we observed no discernible effect of DT on inhibition.

Our results are compatible with the DOH,^16,43^ as in the early stages, when dopaminergic neurons have partially degenerated, the DT impairs inhibitory control. In the H&Y3 stage, when dopamine circuitry depletion is more extensive, the DT does not overdose dopaminergic circuitry and slightly improves both domains of motor inhibition. Although the ON and the OFF conditions do not significantly differ, the DT enhanced reactive inhibition and decreased the SSRT by ∼10 ms in H&Y3 patients (Table 1). Proactive inhibition improves with DT across two of three approaches. At the individual level, there was a higher occurrence of a context effect among H&Y3 patients in the ON compared with the OFF condition (see Fig. 3E, Table 6). At the population level, the cumulative distribution of MTs for H&Y3 patients in the ON condition skews more towards the left side in contrast than for patients in the OFF condition (see Fig. 4E and F), implying a more uniform context effect.

The DOH relies on the idea that in the first stages, the DT overdoses the more intact dopamine circuitries of the ventral striatum impairing cognitive functions relying on them.^16,43^ However, the neural underpinnings of the observed phenomena are certainly more complex. First, we found that reactive inhibition is severely impaired in H&Y1 patients in ON condition. Several studies show that dopamine receptor availability in the dorsal but not in the ventral striatum positively correlates with reactive inhibition.^5-7^ As the dopaminergic nigrostriatal projections to the dorsal striatum are the first to degenerate, the DT should not overdose them. A possible explanation is that the DT overdoses the mesocortical neurons of the ventral tegmental area that project to frontal regions^44^ known to be involved in reactive inhibitory control, such as the precentral gyrus,^45^ the pre-supplementary motor area^46,47^ and the dorsal premotor cortex.^48^ Furthermore, observing enhanced proactive inhibition in H&Y1 and impaired proactive inhibition in H&Y2 patients might sound odd to the DOH since the impairment occurs in a later stage of Parkinson’s disease. However, this could be explained by considering that the whole brain functioning is less affected in the first stages than later on. Since proactive control is a highly demanding strategy, requiring many cognitive resources to actively maintain in the working memory the goal representations and the features of the current context,^49^ likely a more cognitively intact brain can initially compensate for the dopaminergic imbalance. Nevertheless, such compensation cannot occur with Parkinson’s disease progression, and circuits subserving proactive inhibition become overdosed.

Our results are very different from previous ones.^11,12,22,30,31,50-53^ As already stated in the introduction, some features of some studies, such as the low number of participants, the violation of the basic assumption of the horse-race model, the lack of counterbalancing the tests in the ON and OFF states, or grouping Parkinson’s disease patients at different H&Y stages pose severe limitations to the interpretations of the results. A study by Manza *et al*.^30^ is an exception, as only early-stage patients were recruited. The authors found that DT, on average, improved reactive inhibition. However, 4/17 patients showed a reversed effect of DT, and 3/17 did not show any effect. As no effect size measures or BF_10_ values were provided, it is impossible to assess the strength of the results. One relevant difference between this study and ours is that all patients were treated only with L-dopa and/or inhibitors of dopamine catabolism. By contrast, in our sample, some patients also took DA. Nevertheless, our results are independent of the different types of dopaminergic medications. Given that our sample is 40% larger than that of Manza *et al*.,^30^ the large effect sizes and BF_10_ values consistently support our findings, we believe our evidence is robust. Finally, Kübler *et al*.^54^ also addressed the effect of DT on inhibitory control by contrasting the performance in a Go/no-Go task of young- versus late-onset Parkinson’s disease patients with no notable disparity in disease duration or other demographic and clinical features of relevance. They found that inhibition, indexed by the commission error rate, was compromised for young-onset Parkinson’s disease patients in the ON condition compared with the OFF condition, while the opposite was observed for late-onset Parkinson’s disease patients. The authors interpreted this evidence in light of the fact that the pattern of degeneration of dopaminergic neurons is different in these two groups, so that just young-onset Parkinson’s disease patients undergo a DT overdose effect. Further studies are needed to establish the extent to which these results can be overlapped with ours.

### Limitations

One primary limitation of this study is the absence of an assessment of the impact of neurotransmitters other than dopamine on motor inhibition. Previous research has demonstrated that noradrenergic and serotoninergic systems regulate this executive function because atomoxetine,^23-25^ a noradrenaline reuptake inhibitor, and citalopram,^25,26^ a serotonin reuptake inhibitor, enhance reactive inhibition. Therefore, future investigations should examine whether these effects depend on the stage of the disease and extend to proactive inhibition. Conducting such studies is crucial because these drugs could counteract the motor inhibition deficits caused by DT in the early stages of Parkinson’s disease. Another potential limitation pertains to the inclusion of patients already in the disease; i.e. we adopted a cross-sectional rather than a longitudinal approach. Future longitudinal studies on drug-naïve patients must explore the progression of impairments in inhibitory control throughout Parkinson’s disease and evaluate the impact of different dopaminergic drugs.

## Conclusion

This study provides a comprehensive assessment of the effects of DT on motor inhibition across different stages of Parkinson’s disease. We found that the DT has a stage-dependent impact on motor inhibition. As hypothesized by the DOH,^16^ DT selectively impairs inhibition in the early stages of the disease, weakening reactive inhibition in H&Y1 patients and proactive inhibition in H&Y2 patients. In the H&Y3 stage, DT does not overdose dopaminergic circuitry, and it even exerts a slightly beneficial effect on proactive inhibition. Thus, our findings hold significant clinical importance, emphasizing the necessity for careful titration of DT during the initial stages to minimize the interference with inhibitory control while optimizing the reduction of motor symptoms. Additionally, our study supports the idea that inhibitory proficiency can serve as a valuable marker of disease progression,^55^ provided that measures are taken in the OFF state.

## Supplementary Material

fcad350_Supplementary_Data

## Data Availability

Data (after de-identification, in compliance with privacy laws) and the analysis files are freely available from the Open Science Framework platform at the https://osf.io/62kng/.
