# Supplementary material for: Effects of dopaminergic treatment on inhibitory control differ across Hoehn and Yahr stages of Parkinson’s disease
Source: Brain Commun. 2023 Dec 20;6(1):fcad350. doi: 10.1093/braincomms/fcad350 (PMC10757450; doi:10.1093/braincomms/fcad350)
Supplement: fcad350_Supplementary_Data [file fcad350_supplementary_data.docx]

**Supplementary material for:**

**“Effects of dopaminergic treatment on inhibitory control differ across Hoehn and Yahr stages of Parkinson's disease”**

**Table of Contents**

[1. Results of the bibliographic search concerning the effect of dopaminergic therapy on response inhibition 2](#_Toc134094168)

[2. Demographic and clinical features of participants 4](#_Toc134094170)

[3. Statistical analysis on demographic and clinical features 6](#_Toc134094171)

[4. Estimate of the stop-signal reaction time with the integration method 9](#_Toc134094172)

[5. Assessment of race-model assumption and experimental procedures 11](#_Toc134094173)

[5.1 Verification of race model assumption 11](#_Toc134094174)

[5.2. Staircase algorithm performance across groups 12](#_Toc134094175)

[6. Effects of types of dopaminergic medications on the stop signal reaction times, reaction times, and movement times 13](#_Toc134094176)

[7. Correlations between clinical scores of motor severity and behavioral parameters 14](#_Toc134094177)

[8. Complete results of the ANOVA on the stop signal reaction times, reaction times, and movement times 16](#_Toc134094178)

[References 19](#_Toc134094179)

## 1. Results of the bibliographic search concerning the effect of dopaminergic therapy on response inhibition

**Supplementary Figure 1. Flowchart of literature search on the effect of dopaminergic therapy on inhibitory control.**


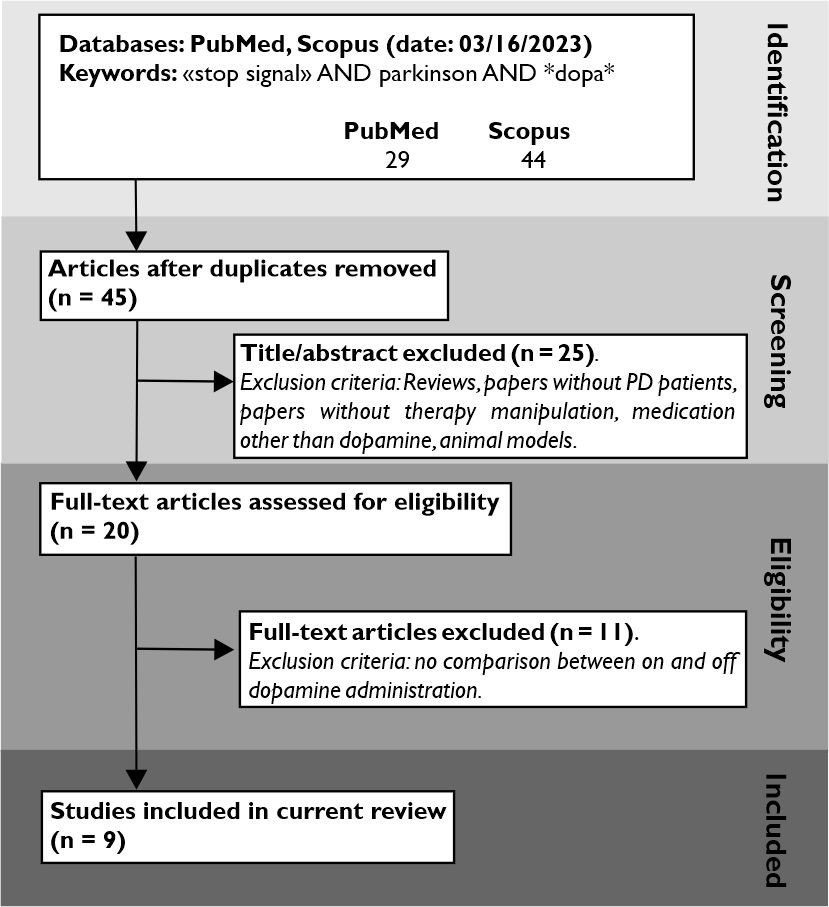


The search was done following the PRISMA guidelines (Page et al., 2021) and yielded a total of 45 unique reports. We examined the full text for eligibility whenever an article could not be excluded based on its title or abstract. The inclusion criteria were as follows: i) the recruitment of Parkinson Disease (PD) patients; ii) the assessment of inhibitory control proficiency under pharmacological treatment (ON) and after an overnight wash-out of at least 12 hours (OFF); iii) the use of the stop-signal task (SST) or its variations. Nine publications satisfied the inclusion criteria. Two employed a variant of the SST (the stop change-SST (Wylie et al., 2018) and the conditional SST (Swann et al., 2009)). The literature search was conducted in March 2023.

**Supplementary Table 1. Summary of the studies identified by the systematic literature research. The table shows the characteristic of the patients with Parkinson’s disease, where applicable healthy control participants, and the main findings of each study.**

| **Effect of dopaminergic replacement therapy on the stop signal reaction time** | | | | | | | | | | | | |
| --- | --- | --- | --- | --- | --- | --- | --- | --- | --- | --- | --- | --- |
| **Authors (Year)** | **Number of participants**  **(Age, years)** | | **H&Y (range)** | **Disease onset (years)** | **Therapy** | **LEDD tot (mg/die)** | **MDS-UPDRS-III**  **OFF- ON** | **TASK** | **Counterbalance** | **Staircase** | **Effect of dopaminergic therapy on reactive inhibition (SSRT)** | **Reacitve inhibition (SSRT)**  **PD vs. HC** |
|  | **PD** | **HC** |  |  |  |  |  |  |  |  |  |  |
| Obeso et al^1^ | 17 (69.4) | 16 (65.7) | 2.1 (mean) | 9.5 | L-DOPA (14) DA&L-DOPA (3) | 915.9 | 14.5 | Conditional SST | Yes | Yes | No effect | HC <PD ON and OFF |
| Alegre et al^2^ | 10 (62.6) | NA | NR | 12.6 | NR | 1207.4 | 12.9 | SST (arrow) | No (First OFF) | Yes | No effect | NA |
| George et al^3^ | 16 (62.6) | 16 (63.5) | 2-3 | 4.94 | L-DOPA (1)  DA&L-DOPA (12)  DA (3) | NR | 7.8 | SST (bimanual) | Yes | Yes | No effect | No effect |
| Cerasa et al^4^ | 12 (67) | NA | 2-2.5 | 5.2 | DA&L-DOPA (12) | 380 | 6.9 | SST (arrow) | No (First OFF) | NR | No effect | NA |
| Picazio et al^5^ | 14 (70.4) | 10 (68.3) | NR | 7.7 | NR | 566.6 | 12.2 | SST (bimanual, arrow) | Yes | Yes | No effect | No effect |
| Wylie et al^6^ | 33 (63.5) | 21 (61.5) | 1-3 | 5 | L-DOPA (14) DA&L-DOPA (12)  DA (7) | 698 | 12.6 | SST Change (bimanual, arrow) | Yes | Yes | PD OFF<PD ON | HC=PD ON; HC< PD OFF |
| Manza et al^7^ | 17 (61.1) | 18 (65.3) | 1-2 | 2.9 | L-DOPA (17) | 476.5 | 6.3 | SST | Yes | Yes | PD OFF<PD ON | HC=PD ON; HC< PD OFF |
| Choudhury et al^8^ | 22 (66.5) | NA | 1-3 | 6.5 | NR | NR | NR | SST | No (First OFF) | No | PD OFF<PD ON | NA |
| **Effect of dopamine agonist replacement therapy on the stop signal reaction time** | | | | | | | | | | | | |
| Claassen et al^9^ | 12 (60.8) | 12 (58.5) | 1 or 2 | 6.1 | Mixed (7) Agonist only (5) | 520.3 | NR | SST (bimanual, arrow) | Yes | Yes | No effect | No effect |

## All values represent means unless otherwise indicated. The columns ‘Task’, ’Counterbalance’, and ’staircase’ report the main features of the stop signal task (SST) version. Bimanual: both hands were used. Arrow: the go-signal was an arrow. Counterbalance: the order of task conditions was counterbalanced across participants (Yes/No). The column ‘H&Y’ indicates the stage of Hoehn & Yahr (H&Y), assessed under pharmacological treatment (ON). The column ‘Therapy’ indicates the type of drugs received. The column ‘MDS-UPDRS-III OFF-ON’ indicates the differences in the total score of the Movement Disorder Society-Unified Parkinson’s Disease Rating Scale part 3 (MDS-UPDRS-III) after an overnight wash-out of at least 12 hours (OFF) and in an ON state. Abbreviations. Dopaminergic therapy: only levodopa (L-DOPA), dopamine agonists (DA), and levodopa plus dopamine agonists (DA & L-DOPA). Total levodopa equivalent daily dose (LEDD tot), levodopa equivalent daily dose of dopamine agonists (LEDD DA). Stop signal reaction time (SSRT). Healthy controls (HC), Parkinson’s disease patients (PD). NR not reported, NA not available

## 2. Demographic and clinical features of participants

To make the reading easy, throughout the document, the groups H&Y 1-1.5, H&Y 2, and H&Y 2.5-3 will be labeled as H&Y1, H&Y2, and H&Y3, respectively.

The demographic and clinical characteristics of each patient are shown in Supplementary Table 2.

**Supplementary Table 2.** **Clinical data of Parkinson’s disease patients.**

| **Patient** | **H&Y**  **(med.)** | **Age**  **(years)** | **Sex**  **(F/M)** | **Handed- ness** | **Education**  **(years)** | **MMSE** | **Years since**  **diagnosis** | **Onset Side**  **(R/L)** | **LEDD tot**  **(mg)** | **LEDD DA**  **(mg)** | **MDS-UPDRS-III**  **(ON)** | **MDS-UPDRS-III**  **(OFF)** |
| --- | --- | --- | --- | --- | --- | --- | --- | --- | --- | --- | --- | --- |
|  |  |  |  |  |  |  |  |  |  |  |  |  |
| **1** | 1 | 72 | M | 90 | 8 | 29 | 2 | L | 520 | 120 | 12 | 20 |
| **2** | 1 | 63 | M | 80 | 8 | 27 | 3 | R | 325 | 225 | 3 | 6 |
| **3** | 1.5 | 60 | F | 100 | 13 | 25 | 2 | L | 670 | 220 | 26 | 38 |
| **4** | 1.5 | 59 | M | 100 | 8 | 30 | 3 | L | 260 | 160 | 8 | 20 |
| **5** | 1.5 | 72 | M | 100 | 8 | 25 | 1 | R | 200 | 0 | 8 | 17 |
| **6** | 1 | 54 | M | 80 | 18 | 30 | 7 | L | 260 | 160 | 7 | 11 |
| **7** | 1 | 50 | M | -50 | 10 | 29 | 6 | L | 560 | 160 | 9 | 11 |
| **8** | 1 | 53 | M | 100 | 8 | 30 | 3 | R | 270 | 120 | 10 | 16 |
| **9** | 1 | 66 | F | 80 | 13 | 30 | 4 | L | 100 | 0 | 7 | 13 |
| **10** | 1.5 | 61 | M | 63.6 | 13 | 30 | 3 | R | 325 | 225 | 10 | 12 |
| **11** | 1.5 | 69 | M | 90 | 13 | 27 | 1.5 | L | 37,5 | 37,5 | 10 | 18 |
| **12** | 1 | 63 | F | 100 | 13 | 30 | 4 | L | 625 | 225 | 4 | 6 |
| **13** | 1 | 53 | F | 100 | 13 | 30 | 3 | R | 250 | 0 | 15 | 23 |
| **14** | 1 | 58 | M | 70 | 8 | 29 | 8 | L | 560 | 160 | 9 | 16 |
| **15** | 1 | 56 | M | 55.6 | 13 | 28 | 3 | R | 220 | 120 | 12 | 15 |
| **16** | 1 | 65 | F | 100 | 8 | 28 | 5 | L | 475 | 75 | 15 | 22 |
| **17** | 1.5 | 60 | M | 100 | 18 | 28 | 1 | L | 250 | 150 | 12 | 18 |
| **18** | 1 | 56 | M | 100 | 8 | 30 | 6 | R | 220 | 120 | 2 | 4 |
| **19** | 1 | 70 | M | 76.5 | 18 | 30 | 3 | R | 450 | 0 | 10 | 15 |
| **20** | 1 | 70 | M | 100 | 11 | 29 | 3 | R | 300 | 0 | 9 | 15 |
|  |  |  |  |  |  |  |  |  |  |  |  |  |
|  | **1.1** | **61.5** | **5/15** | **81.8** | **11.5** | **28.7** | **3.6** | **9/11** | **343.9** | **113.9** | **9.9** | **15.8** |
|  | **(±0.2)** | **(±6.8)** |  | **(±34.0)** | **(±3.6)** | **(±1.6)** | **(±1.9)** |  | **(±175.1)** | **(±82.8)** | **(±5.1)** | **(±7.4)** |
|  |  |  |  |  |  |  |  |  |  |  |  |  |
|  |  |  |  |  |  |  |  |  |  |  |  |  |
| **21** | 2 | 75 | M | 80 | 8 | 25 | 7 | R | 400 | 0 | 23 | 31 |
| **22** | 2 | 72 | M | 100 | 8 | 25.3 | 5 | R | 375 | 75 | 8 | 17 |
| **23** | 2 | 58 | F | 100 | 13 | 28 | 4 | L | 350 | 50 | 20 | 27 |
| **24** | 2 | 47 | M | 100 | 13 | 29 | 5 | R | 375 | 75 | 19 | 25 |
| **25** | 2 | 53 | M | 100 | 8 | 27 | 1.5 | R | 300 | 0 | 20 | 32 |
| **26** | 2 | 62 | M | 80 | 8 | 29 | 10 | L | 450 | 150 | 9 | 15 |
| **27** | 2 | 69 | M | 100 | 8 | 24.2 | 6 | R | 200 | 50 | 24 | 33 |
| **28** | 2 | 69 | M | 100 | 13 | 29 | 3 | L | 180 | 80 | 11 | 31 |
| **29** | 2 | 56 | F | 100 | 13 | 30 | 2 | L | 150 | 0 | 24 | 32 |
| **30** | 2 | 68 | F | 100 | 5 | 27 | 10 | L | 150 | 0 | 11 | 19 |
| **31** | 2 | 61 | F | 90 | 13 | 27 | 2 | R | 400 | 0 | 14 | 28 |
| **32** | 2 | 56 | M | 90 | 13 | 27 | 3 | R | 625 | 225 | 19 | 28 |
| **33** | 2 | 60 | M | 63.6 | 13 | 30 | 3.5 | R | 350 | 0 | 25 | 35 |
| **34** | 2 | 50 | M | 90 | 13 | 28 | 6 | R | 350 | 100 | 32 | 48 |
| **35** | 2 | 67 | M | 100 | 18 | 24.2 | 5 | L | 550 | 0 | 8 | 29 |
| **36** | 2 | 63 | M | 100 | 13 | 24 | 5 | R | 500 | 0 | 24 | 32 |
| **37** | 2 | 64 | M | 100 | 13 | 25.2 | 7 | R | 737.5 | 37.5 | 20 | 30 |
| **38** | 2 | 50 | F | 90 | 13 | 24 | 8 | R | 910 | 300 | 16 | 47 |
| **39** | 2 | 80 | M | 70 | 5 | 26 | 10 | R | 675 | 75 | 22 | 32 |
| **40** | 2 | 50 | F | 100 | 18 | 30 | 1.5 | R | 225 | 75 | 12 | 22 |
|  |  |  |  |  |  |  |  |  |  |  |  |  |
|  | **2** | **61.5** | **6/14** | **92.7** | **11.5** | **26.9** | **5.2** | **14/6** | **395.5** | **66.1** | **18.1** | **29.7** |
|  |  | **(±9.1)** |  | **(±11.1)** | **(±3.7)** | **(±2.1)** | **(±2.8)** |  | **(±194.9)** | **(±83)** | **(±6.7)** | **(±8.2)** |
|  |  |  |  |  |  |  |  |  |  |  |  |  |
|  |  |  |  |  |  |  |  |  |  |  |  |  |
| **41** | 3 | 76 | F | 100 | 5 | 29 | 11 | R | 700 | 150 | 21 | 38 |
| **42** | 3 | 71 | F | 100 | 5 | 29 | 5 | L | 650 | 225 | 12 | 17 |
| **43** | 3 | 70 | M | 100 | 8 | 28 | 4 | R | 1025 | 75 | 40 | 60 |
| **44** | 3 | 83 | M | 70 | 13 | 28 | 9 | L | 300 | 0 | 36 | 36 |
| **45** | 3 | 57 | M | 90 | 13 | 27 | 17 | L | 930 | 80 | 16 | 37 |
| **46** | 3 | 82 | F | 100 | 13 | 28 | 7 | R | 800 | 0 | 29 | 43 |
| **47** | 2.5 | 75 | F | 100 | 5 | 24 | 6 | R | 450 | 0 | 20 | 33 |
| **48** | 3 | 70 | F | 100 | 8 | 25 | 6 | L | 600 | 0 | 24 | 46 |
| **49** | 3 | 76 | M | 100 | 8 | 29 | 17 | R | 780 | 80 | 38 | 49 |
| **50** | 3 | 63 | M | 100 | 13 | 29 | 10 | R | 620 | 120 | 19 | 27 |
| **51** | 3 | 65 | M | 90 | 17 | 30 | 4 | R | 420 | 120 | 20 | 29 |
| **52** | 2.5 | 47 | M | 80 | 13 | 30 | 12 | R | 1375 | 75 | 30 | 45 |
| **53** | 3 | 72 | F | 100 | 5 | 25 | 12 | R | 820 | 120 | 22 | 48 |
| **54** | 3 | 71 | M | 100 | 8 | 25.4 | 10 | R | 400 | 0 | 12 | 28 |
| **55** | 2.5 | 80 | M | 100 | 18 | 30 | 5 | B^a^ | 300 | 0 | 28 | 39 |
| **56** | 2.5 | 65 | M | 100 | 13 | 25 | 11 | R | 1000 | 0 | 10 | 43 |
| **57** | 2.5 | 64 | F | 100 | 8 | 24 | 6 | L | 725 | 75 | 33 | 45 |
| **58** | 2.5 | 67 | M | 100 | 5 | 24 | 6 | R | 400 | 0 | 19 | 34 |
| **59** | 2.5 | 70 | M | 80 | 8 | 29 | 3 | R | 420 | 120 | 13 | 18 |
| **60** | 2.5 | 72 | F | 100 | 5 | 28.3 | 16 | R | 1000 | 37.5 | 9 | 19 |
|  |  |  |  |  |  |  |  |  |  |  |  |  |
|  | **2.8** | **69.8** | **8/12** | **95.5** | **9.6** | **27.3** | **8.9** | **14/5** | **685.8** | **65.3** | **22.6** | **36.7** |
|  | **(±0.2)** | **(±8.5)** |  | **(±8.9)** | **(±4.2)** | **(±2.2)** | **(±4.4)** |  | **(±286.5)** | **(±66.4)** | **(±9.5)** | **(±11.3)** |
|  |  |  |  |  |  |  |  |  |  |  |  |  |
|  |  |  |  |  |  |  |  |  |  |  |  |  |
|  |  |  |  |  |  |  |  |  |  |  |  |  |

For each Parkinson’s disease (PD) patient, the Hoehn & Yahr (H&Y) stage, age, sex, handedness(Oldfield, 1971), years of education, Mini-mental state examination (MMSE) score, years since diagnosis, the onset of PD symptoms, total levodopa equivalent daily dose (LEDD tot), levodopa equivalent daily dose of dopamine agonists (LEDD DA), the total score of the Movement Disorder Society-Unified Parkinson’s Disease Rating Scale part 3 under pharmacological treatment (MDS-UPDRS-III, ON), total MDS-UPDRS-III score after an overnight washout of at least 12 hours (MDS-UPDRS-III, OFF) are given.

^a^This patient had a bilateral onset (B).

## 3. Statistical analysis on demographic and clinical features

Supplementary Tables 3 and 4 show the statistics for demographic and clinical characteristics. Age differences were evaluated via a one-way analysis of variance (ANOVA) with Group as a factor (HC, H&Y1, H&Y2, H&Y3). We found only a significant main effect of Group, as H&Y3 were older than the other groups. A Chi-square independence test showed that groups did not differ in gender proportions. The laterality quotient assessed by the Edinburgh Handedness Inventory (Oldfield, 1971) and the years of Education were not normally distributed. Thus, we checked for differences among Groups via one-way Kruskal-Wallis rank sum tests. The laterality quotient was not different between groups, but years of Education were different. Post hoc Dunn’s tests revealed that healthy controls (HCs) had a longer education than H&Y3 patients.

Regarding clinical characteristics of PD patient groups, a one-way Kruskal-Wallis rank sum test on the Mini-mental state examination scores showed a main effect of the Group because H&Y1 patients had a higher value than H&Y2 patients. A Chi-square independence test showed no differences in the side-of-onset frequencies. The H&Y3 group patients had the earliest disease onset and took the highest total levodopa equivalent daily dose (LEDD tot). Also, the amount of dopamine agonists’ LEDD differed because H&Y1 patients took a higher dose of dopamine agonists, with respect to H&Y2 and H&Y3 patients. However, this difference did not survive the post hoc tests.

The differences in the MDS-UPDRS-III scores were evaluated via a two-way ANOVA (Supplementary Table 4). We found a significant main effect of Treatment because, as expected, motor symptoms worsened in the OFF condition overall. We also found a main effect of Group, which was qualified by the significant interaction Group*Treatment. Post hoc tests revealed that H&Y1 patients always had the lowest score, both in ON and OFF conditions, with respect to H&Y2 and H&Y3 patients. However, the MDS-UPDRS-III of H&Y2 and H&Y3 patients was not statistically different, regardless of the medication condition.

**Supplementary Table 3. Results of the statistical analysis on** **demographic and clinical features of participants.**

| **Measured (dependent) variable with statistical analysis in parentheses** | **Pairwise comparisons** | **Value of parameters** | | **p-values** | |
| --- | --- | --- | --- | --- | --- |
| **Age**  (one-way ANOVA; between-participants factor: Group [HC, H&Y1, H&Y2, H&Y3] | |  |  |  |  |
| **Main effect of Group** |  |  | *F*[3,86] = 5,62 |  | **p<0.001** |
| ***Post hoc tests*** | H&Y3 vs. HC |  | *t(86)* = 3.75 |  | **p<0.002** |
|  | H&Y3 vs. H&Y1 |  | *t(86)* = -3.18 |  | **p=0.012** |
|  | H&Y3 vs. H&Y2 |  | *t(86)* = -3.18 |  | **p=0.012** |
| **Gender**  (Chi-Square Test for Homogeneity) |  |  | *χ^2^(3) = 7*.60 |  | p=0.055 |
| **Handedness**  (one-way Kruskal-Wallis rank sum test; between-participants factors: Group [HC, H&Y1, H&Y2, H&Y3]) | |  | *χ^2^(3) =* 4.52 |  | p=0.210 |
| **Education**  (one-way Kruskal-Wallis rank sum test; between-participants factors: Group [HC, H&Y1, H&Y2, H&Y3]) | |  | *χ^2^(3) =* 7.95 |  | **p=0.047** |
| ***Post hoc (Dunn's Test)*** | H&Y3 vs HC |  | *z = 2.82* |  | **p=0.029** |
| **MMSE**  (one-way Kruskal-Wallis rank sum test; between-participants factor: Group [H&Y1, H&Y2, H&Y3]) | |  | *χ^2^(2) =* 7.80 |  | **p=0.020** |
| ***Post hoc (Dunn's Test)*** | H&Y1 vs. H&Y2 |  | *z = -2.64* |  | **p=0.025** |
|  | H&Y1 vs. H&Y3 |  | *z = -2.11* |  | p=0.104 |
|  | H&Y2 vs. H&Y3 |  | *z = 0.53* |  | p=1 |
| **Onset Side**  (Chi-Square Test for Homogeneity) |  |  | *χ^2^(2) = 4.12* |  | p=0.128 |
| **Years since diagnosis**  (one–way ANOVA; between-participants factor: Group [H&Y1, H&Y2, H&Y3]) | |  | *F*[2,57] =14.31 |  | **p<0.001** |
| ***Post hoc tests (pairwise comparisons)*** | H&Y1 vs. H&Y2 |  | *t(57)* = -1.64 |  | p=0.322 |
|  | H&Y1 vs. H&Y3 |  | *t(57)* = -5.23 |  | **p<0.001** |
|  | H&Y2 vs. H&Y3 |  | *t(57)* = -3.59 |  | **p=0.002** |
| **LEDD tot**  (one–way ANOVA; between-participants factor: Group [H&Y1, H&Y2, H&Y3]) | |  | *F*[2,57] =12.69 |  | **p<0.001** |
| ***Post hoc tests (pairwise comparisons)*** | H&Y1 vs. H&Y2 |  | *t(57)* = -0.96 |  | p=1 |
|  | H&Y1 vs. H&Y3 |  | *t(57)* = -4.76 |  | **p<0.001** |
|  | H&Y2 vs. H&Y3 |  | *t(57)* = -3.80 |  | **p=0.001** |
| **LEDD** **DA**  (one-way Kruskal-Wallis rank sum test; between-participants factor: Group [H&Y1, H&Y2, H&Y3])^a^ | |  | *χ^2^(2) = 6.07* |  | p=0.058 |
|  | |  |  |  |  |

Statistically significant results are reported in bold. All Post hoc tests (pairwise comparisons) had an adjusted alpha level corrected according to Bonferroni. Abbreviations. Analysis of variance (ANOVA). Healthy controls (HC). Hoehn & Yahr (H&Y) groups 1-1.5, 2, and 2.5-3 (H&Y1, H&Y2, and H&Y3). Gender: male and female (M, F). Mini-mental state examination (MMSE). Total levodopa equivalent daily dose (LEDD tot). Levodopa equivalent daily dose of dopamine agonists (LEDD DA). Total score of the Questionnaire for Impulsive-Compulsive Disorders in Parkinson’s Disease–Rating Scale (QUIP-RS).

^a^No significant post hoc tests after Bonferroni correction.

**Supplementary Table 4. Results and statistical parameters of the two-way mixed-design ANOVA on MDS-UPDRS-III scores.**

| **Two-way ANOVA on MDS-UPDRS-III Between-Participants Factors: Group (**H&Y1, H&Y2, H&Y3**); Within-Participant Factor: Treatment (**ON, OFF**)** | | | | | | | |
| --- | --- | --- | --- | --- | --- | --- | --- |
|  |  | **Value of parameters** | ***p-*values** | **M_diff_** | **95% CI** | **Effect Size** | **BF_10_** |
| Main effect: | **Group** | F[2,57] = 24.45 | **p < 0.001** | - | - | ηₚ² = 0.46 | >100 |
| *Post Hoc tests:* | *H&Y1 vs. H&Y2* | t(57) = -4.51 | **p < 0.001** | -11.0 | [-14.7, -7.3] | d = 1.33 | >100 |
|  | *H&Y1 vs. H&Y3* | t(57) = -6.88 | **p < 0.001** | -16.8 | [-21.3, -12.2] | d = 1.66 | >100 |
|  | *H&Y2 vs. H&Y3* | t(57) = -2.37 | p = 0.064 | -5.8 | [-10.7, -0.8] | d = 0.52 | 2.34 |
| Main effect: | **Treatment** | F[1,57] = 187.04 | **p < 0.001** | -10.6 | [-14.5, -6.6] | ηₚ² = 0.77 | >100 |
| Interaction: | **Group x Treatment** | F[2,57] = 9.99 | **p < 0.001** | - | - | ηₚ² = 0.26 | >100 |
| *Post Hoc tests:* | *H&Y1 OFF vs. H&Y1 ON* | t(57) = 4.42 | **p = 0.001** | 5.9 | [4.5, 7.3] | d = 1.91 | >100 |
|  | *H&Y2 OFF vs. H&Y2 ON* | t(57) = 8.68 | **p < 0.001** | 11.6 | [8.7, 14.5] | d = 1.88 | >100 |
|  | *H&Y3 OFF vs. H&Y3 ON* | t(57) = 10.59 | **p < 0.001** | 14.2 | [10.5, 17.8] | d = 1.84 | >100 |
|  | *H&Y1 OFF vs. H&Y2 OFF* | t(57) = -4.80 | **p < 0.001** | -13.8 | [-18.8, -8.9] | d = 1.77 | >100 |
|  | *H&Y1 OFF vs. H&Y3 OFF* | t(57) = -7.24 | **p < 0.001** | -20.9 | [-27.0, -14.8] | d = 2.19 | >100 |
|  | *H&Y2 OFF vs. H&Y3 OFF* | t(57) = -2.44 | p = 0.266 | -7.1 | [-13.4, -0.7] | d = 0.71 | 2.17 |
|  | *H&Y1 ON vs. H&Y2 ON* | t(57) = -3.53 | **p = 0.012** | -8.2 | [-12.0, -4.3] | d = 1.37 | >100 |
|  | *H&Y1 ON vs. H&Y3 ON* | t(57) = -5.48 | **p < 0.001** | -12.7 | [-17.6, -7.7] | d = 1.66 | >100 |
|  | *H&Y2 ON vs. H&Y3 ON* | t(57) = -1.95 | p = 0.844 | -4.5 | [-9.8, 0.8] | d = 0.55 | 1.01 |
|  |  |  |  |  |  |  |  |

Statistically significant results are reported in bold. All Post hoc tests (pairwise comparisons) had an adjusted alpha level corrected according to Bonferroni. Bayes factors report the ratio between the null versus the alternative hypothesis (BF10), Effect sizes are reported as partial eta squared (ηp2) and Cohen's d. Abbreviations. Analysis of variance (ANOVA). Movement Disorder Society-Unified Parkinson’s Disease Rating Scale part 3 (MDS-UPDRS-III). Hoehn & Yahr (H&Y) groups 1-1.5, 2, and 2.5-3 (H&Y1, H&Y2, and H&Y3, respectively). Assessment performed under pharmacological treatment (ON), and after an overnight wash-out of at least 12 hours (OFF).

## 4. Estimate of the stop-signal reaction time with the integration method

The stop-signal reaction time (SSRT) is estimated by exploiting the “horse race” model^10^. Such a model assumes that the behavioral outcome of the stop-signal task is the result of a race between the go-process (i.e., the process started by the go-signal eliciting movement initiation) and the stop-process (i.e., the process started by the stop-signal and eliciting movement inhibition). These processes are assumed to be stochastically independent. The race is won by the process that first reaches an arbitrary threshold. In stop trials, if the stop process wins, participants will suppress their response, but if the go-process wins, participants will respond although they should not (stop-failure). The independence assumption implies that the stop trials’ reaction time (RT) distribution (regardless of whether a response is canceled or not) would be the same as the no-stop trials’ RT distribution^10^. In the integration method, SSRT is estimated by subtracting the finishing time of the stop process from the starting time ^10-12^. The former corresponds to the nth RT (with n= the number of RTs in the RT distribution of no-stop trials multiplied by the observed proportion of stop-failure trials or p(failure),^13^ (Supplementary Figure 1). The latter corresponds to the mean stop-signal delay (SSD), i.e., the average time interval between the go-signal and the stop-signal presentation. The mean SSD value is computed using the mid-run estimates method^14^. Briefly, SSD sequences produced by the staircase algorithm consist of increasing or decreasing values (ramps) based on the participants’ performance. For each participant, mean SSDs were computed by averaging SSD values obtained from the midpoints of every second ramp. Finally, the value of the SSRT is obtained by subtracting the length of the nth RT from the mean SSD. The integration method using the staircase algorithm produces the most reliable and least biased non-parametric SSRT estimate, given that it is almost insensitive to assumptions’ violations of the race model^11,13^ and it is resistant to proactive slowing occurs^15^.

**
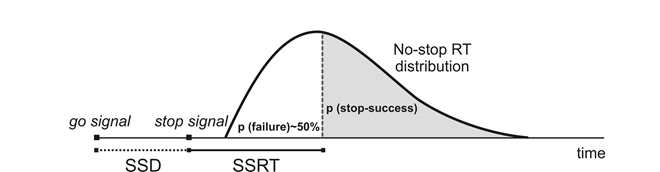
Supplementary Figure 2. Estimate of the stop signal reaction time (SSRT) with the integration method.**

The SSRT is obtained by subtracting the starting time of the stop process, the mean stop-signal delay (SSD), from its finishing time, which is calculated by ‘integrating’ the no-stop trials reaction times (RT) distribution from the onset of the go-signal until the nth RT, with n = the number of RTs in the RT distribution of no-stop multiplied by the proportion of stop-failure trials [p(failure)]. For instance, when there are 360 no-stop trials, and p(failure) is 0.5, the nth RT is the 180th fastest no-stop RT.

## 5. Assessment of race-model assumption and experimental procedures

## 5.1 Verification of race model assumption

We assessed the assumption of independence between the go- and the stop-process by comparing the RTs of no-stop and stop-failure trials^13,16^. When a participant does not cancel a movement in stop trials (stop-failure trials), it is because the go-process has been completed before the stop-process. Thus, the RTs of stop-failure trials have to be shorter than the sum of the SSD (the time delay between the go- and the stop-signal) plus the SSRT (indicating the end of the stop-process, Supplementary Figure 2). As the distribution of stop-failure trials’ RTs partially overlaps with that of the no-stop trials’ RTs, the mean RT of the stop-failure trials should be shorter than that of no-stop trials.

We checked this assumption in two ways. First, we used a within-participant approach. In each participant, we assessed whether the cumulative distribution of stop-failure trials’ RTs was significantly different from that of no-stop trials. In all HCs (n=30) and all patients’ conditions (n=120), the cumulative distributions of stop-failure RTs were shifted to the right with respect to those of no-stop RTs, indicating that the stop-failure RTs were always faster than the no-stop RTs (one-tailed two-sample Kolmogorov-Smirnov tests, all *p*<0.025). Second, using a between-participant approach, we used a three-way mixed-design ANOVA [between-participants factor: Group (H&Y1, H&Y2, H&Y3); within-participants factors: Trial Type (no-stop, stop-failure trials); Treatment (ON, OFF)]. We found a main effect of Trial Type, showing that the average RT of stop-failure trials was faster than that of no-stop trials (Supplementary Table 5). We also found a significant main effect of the Group. Post hoc tests revealed that H&Y2 and H&Y3 patients had shorter RTs than the H&Y1 group. Finally, a two-way mixed-design ANOVA on HCs [between-participants factor: Gender (Male, Female); within-participants factor: Trial Type (no-stop, stop failure trials)] showed only a significant main effect of Trial Type (*F*[1,28] = 216.4; *p*<0.001; *η*_p_^2^ = 0.88), because, again, mean RT of stop-failure trials were shorter than mean RT of no-stop trials. In conclusion, the assumption of independence was always met.

**Supplementary Table 5. Results of the three-way ANOVA on Parkinson’s patients’ RT of stop-failure and no-stop trials**

| **Three-way mixed-design ANOVA on RTs *Between-Participants Factors***: **Group** (H&Y1, H&Y2, H&Y3); ***Within-Participant Factors***: **Trial Type** (no-stop, stop-failure trials), **Treatment** (ON, OFF) | | | | | | | |
| --- | --- | --- | --- | --- | --- | --- | --- |
|  |  | **Value of parameters** | ***p-*values** | **M_diff_** | **95% CI** | **Effect Size** | **BF_10_** |
| Main effect: | **Trial Type** | *F*[1, 57]=534.75 | **p < 0.001** | 115.7 | [107.3, 124.1] | *η*_p_^2^ = *0.90* | >100 |
| Main effect: | **Group** | *F*[2, 57]=6.61 | **p = 0.003** | - | - | *η*_p_^2^ = *0.19* | 14.87 |
| *Post Hoc tests:* | *H&Y1 vs. H&Y2* | t(57) = 2.85 | **p = 0.018** | 81.5 | [47.0, 115.9] | d = 0.74 | >100 |
|  | *H&Y1 vs. H&Y3* | t(57) = 3.38 | **p = 0.004** | 96.5 | [58.8, 134.2] | d = 0.80 | >100 |
|  |  |  |  |  |  |  |  |

Only significant main effects and interactions are shown. All Post hoc tests (pairwise comparisons) had an adjusted alpha level corrected according to Bonferroni. Bayes factors report the ratio between the null versus the alternative hypothesis (BF10). Effect sizes are reported as partial eta squared (ηp2) and Cohen's d. Abbreviations. Analysis of variance (ANOVA). Confidence Interval (CI), Hoehn & Yahr (H&Y) groups 1-1.5, 2, and 2.5-3 (H&Y1, H&Y2, and H&Y3, respectively). Assessment performed under pharmacological treatment (ON), and after an overnight wash-out of at least 12 hours (OFF).

## 5.2. Staircase algorithm performance across groups

It has been shown that SSRT estimates are most reliable when the probability of responding on a stop trial [p(failure)] is close to 0.50^11^. This is why we set the staircase procedure to keep the success rate of stop-signal trials around 50%. Nevertheless, the integration method produces reliable estimates even when p(failure) is lower than 0.25 or higher than 0.75^13,17^. On average (Supplementary Table 1), patients in both conditions and HCs had a p(failure) close to 0.50. The p(failure) range was between 0.45-0.77. Just one patient in one condition had a p(failure)=0.77. Across all cases (n=150), just four times participants had a p(failure)>0.70 (all H&Y3 patients). Therefore, we could state that the staircase algorithm worked very well.

We also assessed differences of p(failure) across groups [between-participants factor: Group (HC, H&Y1, H&Y2, H&Y3)]. To this end, we ran two separate one-way Kruskal-Wallis rank sum tests to compare HCs with patients in ON or OFF conditions. The rank sum test in the ON condition did not show differences between p(failure) across groups (χ2(3) = 7.49, p=0.058). Instead, the rank sum test in the OFF condition revealed a significant difference (χ2(3) = 16.65, p<0.001). Post hoc analyses (Dunn’s test) showed that H&Y1 patients had a lower P(failure) than HCs (z=-2.72; p=0.039) and H&Y3 patients (z=4.01; p<0.001). All other comparisons were not significant.

## 6. Effects of types of dopaminergic medications on the stop signal reaction times, reaction times, and movement times

**Supplementary Table 6. Statistical analysis results of stop-signal reaction times (SSRTs) in Parkinson's groups and healthy controls.**

| **Two-way ANOVA on SSRT Between-Participants Factors: Therapy (L-Dopa, DA, DA & L-Dopa); Within-Participant Factors: Treatment (ON, OFF)** | | | | | | | | | | | | |  |
| --- | --- | --- | --- | --- | --- | --- | --- | --- | --- | --- | --- | --- | --- |
|  |  | | **Value of parameters** | | ***p-*values** | | **M_diff_** | **95% CI** | | **Effect Size** | | **BF_10_** |  |
| Main effect: | **Therapy** | | F[2,57] = 3.22 | | **p = 0.047** | | - | - | | ηₚ² = 0.1 | | 1.21 |  |
| *Post Hoc tests:* | *DA vs L-Dopa* | | t(57) = -2.46 | | p = 0.051 | | -30.24 | [-49.1, -11.4] | | d = -0.75 | | 7.5 |  |
|  | *DA vs DA & L-Dopa* | | t(57) = -2.13 | | p = 0.112 | | -24.73 | [-41.2, -8.2] | | d = -0.65 | | 4.67 |  |
|  | *L-Dopa vs DA & L-Dopa* | | t(57)=0.58 | | p=1 | | 5.51 | [-11.8, 22.8] | | d = 0.13 | | 0.26 |  |
| Main effect: | **Treatment** | | F[1,57] = 4.33 | | **p = 0.042** | | -10.91 | [-0.58, 22.4] | | ηₚ² = 0.71 | | 0.95 |  |
| Interaction: | **Therapy x Treatment** | | F[2,57] = 0.58 | | p = 0.56 | | - | - | | ηₚ² = 0.2 | | 0.21 |  |
|  | | | | | | | | | | | | | |
| **Three-way ANOVA on RT Between-Participants Factors: Therapy (L-Dopa, DA, DA & L-Dopa); Within-Participant Factors: Treatment (ON, OFF); Trial type (No-Stop, Go-Only)** | | | | | | | | | | | | | |
|  | | |  | | **Value of parameters** | | ***p-*values** | **M_diff_** | **95% CI** | **Effect Size** | | **BF_10_** | |
| Main effect: | | | **Therapy** | | F[2,57] = 0.1 | | p = 0.91 | - | - | ηₚ² = 0.13 | | 0.12 | |
| Main effect: | | | **Trial type** | | F[1,57] = 315.4 | | **p<0.001** | -252.8 | [239.8, 285.7] | ηₚ² = 0.85 | | >100 | |
| Main effect: | | | **Treatment** | | F[1,57] = 1.3 | | p=0.26 | 8.96 | [23.9, 6.1] | ηₚ² = 0.02 | | 0.2 | |
| Interaction: | | | **Therapy x Trial type** | | F[2,57] = 2.71 | | p = 0.07 | - | - | ηₚ² = 0.09 | | 5.11 | |
| Interaction: | | | **Therapy x Treatment** | | F[2,57] = 0.08 | | p = 0.92 | - | - | ηₚ² = 0.003 | | 0.09 | |
| Interaction: | | | **Trial type x Treatment** | | F[1,57] = 1.69 | | p = 0.2 | - | - | ηₚ² = 0.02 | | 0.37 | |
| Interaction: | | | **Trial type x Treatment x Therapy** | | F[2,57] = 0.38 | | p = 0.68 | - | - | ηₚ² = 0.013 | | 0.14 | |
|  | | | | | | | | | | | | | |
| **Three-way ANOVA on MT Between-Participants Factors: Therapy (L-Dopa, DA, DA & L-Dopa); Within-Participant Factors: Treatment (ON, OFF); Trial type (No-Stop, Go-Only)** | | | | | | | | | | | | | |
|  | | |  | | **Value of parameters** | | ***p-*values** | **M_diff_** | **95% CI** | **Effect Size** | | **BF_10_** | |
| Main effect: | | | **Therapy** | | F[2,57] = 4.27 | | **p = 0.019** | - | - | ηₚ² = 0.13 | | 3.64 | |
| *Post Hoc tests* | | | *DA vs L-Dopa* | | t(57) = -2.92 | | **p = 0.015** | -180.6 | [-244.3, -116.8] | d = -0.99 | | >100 | |
|  | | | *DA vs DA & L-Dopa* | | t(57) = -1.88 | | p = 0.19 | -110.0 | [-171.5, -48.5] | d = -0.56 | | 16.1 | |
|  | | | *L-Dopa vs DA & L-Dopa* | | t(57)= 1.47 | | p = 0.44 | 70.6 | [13.9, 127.2] | d = 0.35 | | 2.34 | |
| Main effect: | | | **Trial type** | | F[1,57] = 3.2 | | p=0.08 | 36.1 | [10.5, 61.7] | ηₚ² = 0.05 | | 1.94 | |
| Main effect: | | | **Treatment** | | F[1,57] = 0.27 | | p=0.61 | -15.7 | [-47.3, 15.8] | ηₚ² = 0.004 | | 1.99 | |
| Interaction: | | | **Therapy x Trial type** | | F[2,57] = 1.64 | | p = 0.2 | - | - | ηₚ² = 0.05 | | 0.22 | |
| Interaction: | | | **Therapy x Treatment** | | F[2,57] = 0.24 | | p = 0.79 | - | - | ηₚ² = 0.008 | | 0.13 | |
| Interaction: | | | **Trial type x Treatment** | | F[1,57] = 0.07 | | p = 0.8 | - | - | ηₚ² = 0.001 | | 0.26 | |
| Interaction: | | | **Trial type x Treatment x Therapy** | | F[2,57] = 0.33 | | p = 0.72 | - | - | ηₚ² = 0.011 | | 0.15 | |

## All Post hoc tests (pairwise comparisons) had an adjusted alpha level corrected according to Bonferroni. Bayes factors report the ratio between the null versus the alternative hypothesis (BF10). Effect sizes are reported as partial eta squared (ηp2) and Cohen's d. Abbreviations. Analysis of variance (ANOVA). Confidence Interval (CI), Hoehn & Yahr (H&Y) groups 1-1.5, 2, and 2.5-3 (H&Y1, H&Y2, and H&Y3, respectively). Assessment performed under pharmacological treatment (ON), and after an overnight wash-out of at least 12 hours (OFF). Dopaminergic therapy: only levodopa (L-DOPA), dopamine agonists (DA), and levodopa plus dopamine agonists (DA & L-DOPA).

## 7. Correlations between clinical scores of motor severity and behavioral parameters

To assess whether patients’ symptoms severity, measured on the MDS-UPDRS-III, correlates with the behavioral parameters characterizing the stop-signal task (i.e., the SSRTs, the RTs, and MTs of no-stop signal trials), we computed the Spearman’s rank correlation coefficients (Supplementary Figure 3). All parameters correlated with the MDS-UPDRS-III score in both ON and OFF conditions. The SSRTs were positively correlated with the MDS-UPDRS-III values, indicating that the more severe the motor symptoms, the more reactive inhibition is impaired (Supplementary Figure 3A, 3B). Conversely, the RTs of no-stop signal trials were anti-correlated with the MDS-UPDRS-III values, indicating that the more severe the motor symptoms, the faster, i.e., more impulsive, were patients’ responses (Supplementary Figure 3C, 3D). Finally, the MTs of no-stop trials correlated positively with the UPDRS scores (Supplementary Figure 3E, 3F), i.e., the more severe the motor symptoms, the slower the MTs. In conclusion, patients with more severe motor symptoms have a deficit in reactive inhibition, as their SSRTs increased, and in proactive inhibition, as the RTs of no-stop trials are shortened and the MTs are lengthened.

**Supplementary Figure 3 Correlations between behavioral parameters of the stop signal task and the MDS-UPDRS-III scores of Parkinson’s patients.**


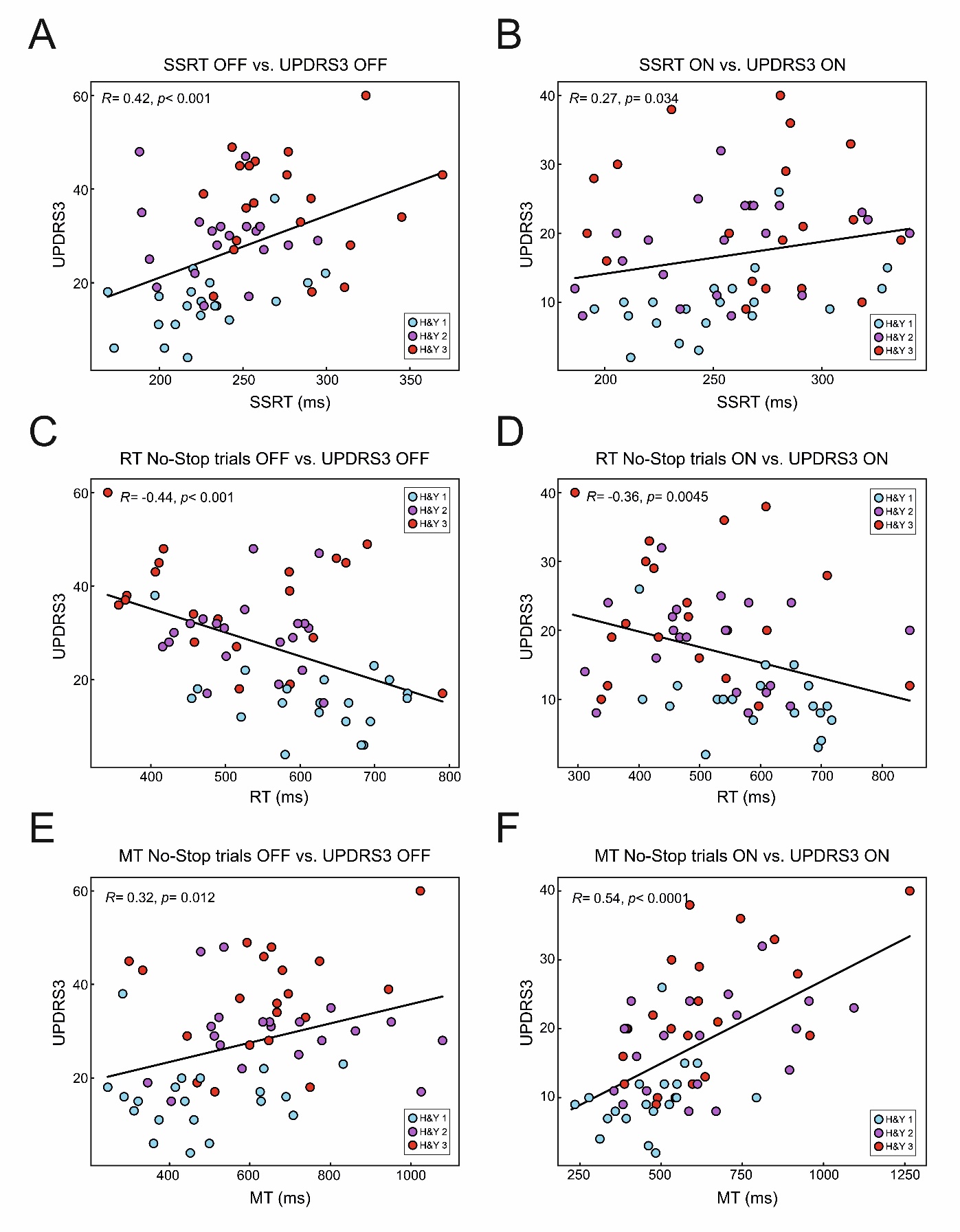


## Scatterplots with regression lines between the Movement Disorder Society-Unified Parkinson’s Disease Rating Scale part 3 (MDS-UPDRS-III) scores and stop-signal reaction times (SSRT) in OFF (A) and ON state (B). (C) Scatterplots with regression lines between no-stop trial reaction times (RTs) in OFF (C) and ON state (D). Scatterplots with no-stop movement times (MT) in OFF (E) and ON state (F). Each dot represents one patient (n=60). Light blue dots represent patients in stages 1-1.5 (H&Y1, n=20); ii) violet dots represent patients in stage 2 (H&Y2, n=20), and iii red dots represent patients in stages 2.5-3 (H&Y3, n=20). Abbreviations: milliseconds (ms). Spearman’s rank coefficient ρ (R). P-values (p).

## 8. Complete results of the ANOVA on the stop signal reaction times, reaction times, and movement times

**Supplementary Table 7. Results of all ANOVA on behavioral parameters.**

| **Two-way ANOVA on SSRT Between-Participants Factors: Group (H&Y1, H&Y2, H&Y3); Within-Participant Factors: Treatment (ON, OFF)** | | | | | | | |
| --- | --- | --- | --- | --- | --- | --- | --- |
|  |  | **Value of parameters** | ***p-*values** | **M_diff_** | **95% CI** | **Effect Size** | **BF_10_** |
| Main effect: | **Group** | F[2,57] = 6.13 | **p = 0.004** | - | - | ηₚ² = 0.18 | 10.38 |
| *Post Hoc tests:* | *H&Y1 vs. H&Y2* | t(57) = -0.66 | p = 1 | -6.5 | [-23.2, 10.2] | d = 0.17 | 0.30 |
|  | *H&Y1 vs. H&Y3* | t(57) = -3.31 | **p = 0.005** | -32.8 | [-50.2, -15.3] | d = 0.84 | 74.14 |
|  | *H&Y2 vs. H&Y3* | t(57) = -2.65 | **p = 0.031** | -26.3 | [-43.6, -8.9] | d = 0.67 | 10.65 |
| Main effect: | **Treatment** | F[1,57] = 3.93 | p = 0.052 | 10.9 | [-0.6, 22.4] | ηₚ² = 0.06 | 1.00 |
| Interaction: | **Group x Treatment** | F[2,57] = 3.61 | **p = 0.033** | - | - | ηₚ² = 0.11 | 1.99 |
| *Post Hoc tests:* | *H&Y1 OFF vs. H&Y1 ON* | t(57) = -2.65 | **p = 0.031** | -25.3 | [-41.3, -9.2] | d = 0.74 | 11.67 |
|  | *H&Y2 OFF vs. H&Y2 ON* | t(57) = -1.78 | p = 0.242 | -17.0 | [-40.9, 7] | d = 0.33 | 0.59 |
|  | *H&Y3 OFF vs. H&Y3 ON* | t(57) = 0.99 | p = 0.976 | 9.5 | [-9.6, 28.5] | d = 0.23 | 0.37 |
|  |  |  |  |  |  |  |  |
| **One-way ANOVA on SSRT in OFF condition Between-Participants Factor: Group (HC, H&Y1, H&Y2, H&Y3)** | | | | | | | |
|  |  | **Value of parameters** | ***p-*values** | **M_diff_** | **95% CI** | **Effect Size** | **BF_10_** |
| Main effect: | **Group** | F[3,86] = 13.49 | **p < 0.001** | - | - | ηₚ² = 0.32 | >100 |
| *Post Hoc tests:* | *HC vs. H&Y1* | t(86) = -0.56 | p = 1 | -5.2 | [-23.4, 13.1] | d = 0.18 | 0.33 |
|  | *HC vs. H&Y2* | t(86) = -1.73 | p = 0.528 | -15.8 | [-32.2, 0.6] | d = 0.58 | 1.45 |
|  | *HC vs. H&Y3* | t(86) = -6.03 | **p < 0.001** | -55.3 | [-75.5, -35.0] | d = 1.75 | >100 |
|  | *H&Y1 vs. H&Y2* | t(86) = -1.06 | p = 1 | -10.7 | [-31.1, 9.8] | d = 0.33 | 0.48 |
|  | *H&Y1 vs. H&Y3* | t(86) = -4.99 | **p < 0.001** | -50.1 | [-73.6, -26.6] | d = 1.37 | >100 |
|  | *H&Y2 vs. H&Y3* | t(86) = -3.93 | **p = 0.001** | -39.5 | [-61.6, -17.3] | d = 1.14 | 34.67 |
|  |  |  |  |  |  |  |  |
| **One-way ANOVA on SSRT in ON condition Between-Participants Factor: Group (HC, H&Y1, H&Y2, H&Y3)** | | | | | | | |
|  |  | **Value of parameters** | ***p-*values** | **M_diff_** | **95% CI** | **Effect Size** | **BF_10_** |
| Main effect: | **Group** | F[3,86] = 7.24 | **p < 0.001** | - | - | ηₚ² = 0.20 | >100 |
| *Post Hoc tests:* | *HC vs. H&Y1* | t(86) = -2.88 | **p = 0.030** | -30.4 | [-50.1, -10.7] | d = 0.98 | 23.78 |
|  | *HC vs. H&Y2* | t(86) = -3.1 | **p = 0.016** | -32.8 | [-54.6, -11.0] | d = 0.98 | 23.41 |
|  | *HC vs. H&Y3* | t(86) = -4.33 | **p < 0.001** | -45.8 | [-67.6, -24.1] | d = 1.37 | >100 |
|  | *H&Y1 vs. H&Y2* | t(86) = -0.2 | p = 1 | -2.4 | [-28.1, 23.4] | d = 0.06 | 0.31 |
|  | *H&Y1 vs. H&Y3* | t(86) = -1.33 | p = 1 | -15.4 | [-41.2, 10.4] | d = 0.38 | 0.55 |
|  | *H&Y2 vs. H&Y3* | t(86) = -1.13 | p = 1 | -13.0 | [-40.4, 14.3] | d = 0.31 | 0.45 |
|  |  |  |  |  |  |  |  |
| **Three-way ANOVA on RT Between-Participants Factors: Group (H&Y1, H&Y2, H&Y3); Within-Participant Factors: Treatment (ON, OFF); Trial type (No-Stop, Go-Only)** | | | | | | | |
|  |  | **Value of parameters** | ***p-*values** | **M_diff_** | **95% CI** | **Effect Size** | **BF_10_** |
| Main effect: | **Group** | F[2,57] = 2.69 | p = 0.076 | - | - | ηₚ² = 0.09 | 0.69 |
| Main effect: | **Treatment** | F[1,57] = 1.23 | p = 0.273 | -8.96 | [-24.0, 6.1] | ηₚ² = 0.02 | 0.21 |
| Main effect: | **Trial type** | F[1,57] = 378.59 | **p < 0.001** | 262.8 | [239.8, 285.7] | ηₚ² = 0.87 | >100 |
| Interaction: | **Group x Treatment** | F[2,57] = 0.22 | p = 0.799 | - | - | ηₚ² = 0.01 | 0.09 |
| Interaction: | **Group x Trial type** | F[2,57] = 6.81 | **p = 0.002** | - | - | ηₚ² = 0.19 | >100 |
| *Post Hoc tests:* | *H&Y1 No-Stop vs. Go-Only* | t(57) = 14.20 | **p < 0.001** | 332.0 | [297.7, 366.3] | d = 3.03 | >100 |
|  | *H&Y2 No-Stop vs. Go-Only* | t(57) = 10.23 | **p < 0.001** | 239.4 | [200.4, 278.3] | d = 1.97 | >100 |
|  | *H&Y3 No-Stop vs. Go-Only* | t(57) = 9.27 | **p < 0.001** | 216.8 | [177.5, 256.2] | d = 1.76 | >100 |
|  | *Go-Only H&Y1 vs. H&Y2* | t(57) = -0.86 | p = 1 | -13.7 | [-38.5, 11.1] | d = 0.25 | 0.39 |
|  | *Go-Only H&Y1 vs. H&Y3* | t(57) = -0.95 | p = 1 | -15.1 | [-41.5, 11.2] | d = 0.26 | 0.41 |
|  | *Go-Only H&Y2 vs. H&Y3* | t(57) = -0.09 | p = 1 | -1.5 | [-28.3, 25.3] | d = 0.02 | 0.23 |
|  | *No-Stop H&Y1 vs. H&Y2* | t(57) = 2.46 | p = 0.151 | 78.9 | [33.6, 124.3] | d = 0.78 | 14.5 |
|  | *No-Stop H&Y1 vs. H&Y3* | t(57) = 3.12 | **p = 0.025** | 100.0 | [47.9, 152.2] | d = 0.85 | 94.76 |
|  | *No-Stop H&Y2 vs. H&Y3* | t(57) = 0.66 | p = 1 | 21.1 | [-31.2, 73.4] | d = 0.18 | 0.31 |
| Interaction: | **Treatment x Trial type** | F[1,57] = 1.98 | p = 0.165 | - | - | ηₚ² = 0.03 | 0.30 |
| Interaction: | **Group x Treatment x Trial type** | F[2,57] = 0.71 | p = 0.495 | - | - | ηₚ² = 0.02 | 0.19 |
|  |  |  |  |  |  |  |  |
| **Two-way ANOVA on RT in OFF condition Between-Participants Factors: Group (HC, H&Y1, H&Y2, H&Y3) Within-Participant Factors: Trial type (No-Stop, Go-Only)** | | | | | | | |
|  |  | **Value of parameters** | ***p-*values** | **M_diff_** | **95% CI** | **Effect Size** | **BF_10_** |
| Main effect: | **Group** | F[3,86] = 1.99 | p = 0.122 | - | - | ηₚ² = 0.07 | 0.33 |
| Main effect: | **Trial type** | F[1,86] = 454.59 | **p < 0.001** | 251.1 | [218, 269.6] | ηₚ² = 0.84 | >100 |
| Interaction: | **Group x Trial type** | F[3,86] = 8.68 | **p < 0.001** | - | - | ηₚ² = 0.23 | >100 |
| *Post Hoc tests:* | *HC No-Stop vs. Go-Only* | t(86) = -9.25 | **p < 0.001** | 185.8 | [146.4, 225.2] | d = 1.76 | >100 |
|  | *H&Y1 No-Stop vs. Go-Only* | t(86) = -14.09 | **p < 0.001** | 346.6 | [298.2, 395.0] | d = 3.35 | >100 |
|  | *H&Y2 No-Stop vs. Go-Only* | t(86) = -9.66 | **p < 0.001** | 237.5 | [186.0, 289.0] | d = 2.16 | >100 |
|  | *H&Y3 No-Stop vs. Go-Only* | t(86) = -9.53 | **p < 0.001** | 234.3 | [177.0, 291.6] | d = 1.91 | >100 |
|  | *Go-Only HC vs. H&Y1* | t(86) = 1.95 | p = 0.867 | 38.6 | [-0.6, 77.8] | d = 0.52 | 1.06 |
|  | *Go-Only HC vs. H&Y2* | t(86) = 0.63 | p = 1 | 12.4 | [-29.0, 53.7] | d = 0.16 | 0.33 |
|  | *Go-Only HC vs. H&Y3* | t(86) = 1.37 | p = 1 | 27.1 | [-14.7, 69] | d = 0.35 | 0.53 |
|  | *Go-Only H&Y1 vs. H&Y2* | t(86) = -1.21 | p = 1 | -26.2 | [-62.1, 9.6] | d = 0.47 | 0.73 |
|  | *Go-Only H&Y1 vs. H&Y3* | t(86) = -0.53 | p = 1 | -11.5 | [-47.9, 25] | d = 0.20 | 0.36 |
|  | *Go-Only H&Y2 vs. H&Y3* | t(86) = 0.68 | p = 1 | 14.8 | [-24.0, 53.6] | d = 0.24 | 0.39 |
|  | *No-Stop HC vs. H&Y1* | t(86) = -4.18 | **p = 0.001** | -122.2 | [-179.9, -64.5] | d = 1.23 | >100 |
|  | *No-Stop HC vs. H&Y2* | t(86) = -1.35 | p = 1 | -39.3 | [-88.1, 9.5] | d = 0.44 | 0.73 |
|  | *No-Stop HC vs. H&Y3* | t(86) = -0.73 | p = 1 | -21.4 | [-90.2, 47.4] | d = 0.19 | 0.34 |
|  | *No-Stop H&Y1 vs. H&Y2* | t(86) = 2.59 | p = 0.181 | 82.9 | [27.3, 138.5] | d = 0.96 | 9.39 |
|  | *No-Stop H&Y1 vs. H&Y3* | t(86) = 3.15 | **p = 0.036** | 100.8 | [27.5, 174.1] | d = 0.88 | 5.79 |
|  | *No-Stop H&Y2 vs. H&Y3* | t(86) = 0.56 | p = 1 | 17.9 | [-49.1, 85.0] | d = 0.17 | 0.35 |
|  |  |  |  |  |  |  |  |
| **Two-way ANOVA on RT in ON condition Between-Participants Factors: Group (HC, H&Y1, H&Y2, H&Y3) Within-Participant Factors: Trial type (No-Stop, Go-Only)** | | | | | | | |
|  |  | **Value of parameters** | ***p-*values** | **M_diff_** | **95% CI** | **Effect Size** | **BF_10_** |
| Main effect: | **Group** | F[3,86] = 1.26 | p = 0.292 | - | - | ηₚ² = 0.04 | 0.14 |
| Main effect: | **Trial type** | F[1,86] = 347.79 | **p < 0.001** | 230.4 | [203.8, 257] | ηₚ² = 0.80 | >100 |
| Interaction: | **Group x Trial type** | F[3,86] = 5.55 | **p = 0.002** | - | - | ηₚ² = 0.16 | 31.66 |
| *Post Hoc tests:* | *HC No-Stop vs. Go-Only* | t(86) = -8.61 | **p < 0.001** | 185.8 | [146.4, 225.2] | d = 1.76 | >100 |
|  | *H&Y1 No-Stop vs. Go-Only* | t(86) = -12.01 | **p < 0.001** | 317.4 | [265.1, 369.8] | d = 2.84 | >100 |
|  | *H&Y2 No-Stop vs. Go-Only* | t(86) = -9.13 | **p < 0.001** | 241.3 | [177.9, 304.7] | d = 1.78 | >100 |
|  | *H&Y3 No-Stop vs. Go-Only* | t(86) = -7.54 | **p < 0.001** | 199.4 | [141.2, 257.5] | d = 1.61 | >100 |
|  | *Go-Only HC vs. H&Y1* | t(86) = 1.58 | p = 1 | 31.6 | [-9.4, 72.6] | d = 0.42 | 0.66 |
|  | *Go-Only HC vs. H&Y2* | t(86) = 1.52 | p = 1 | 30.5 | [-9.2, 70.2] | d = 0.41 | 0.64 |
|  | *Go-Only HC vs. H&Y3* | t(86) = 0.64 | p = 1 | 12.8 | [-30.5, 56.1] | d = 0.16 | 0.33 |
|  | *Go-Only H&Y1 vs. H&Y2* | t(86) = -0.05 | p = 1 | -1.1 | [-37.1, 34.8] | d = 0.02 | 0.31 |
|  | *Go-Only H&Y1 vs. H&Y3* | t(86) = -0.86 | p = 1 | -18.8 | [-58.7, 21.1] | d = 0.30 | 0.44 |
|  | *Go-Only H&Y2 vs. H&Y3* | t(86) = -0.8 | p = 1 | -17.7 | [-56.3, 20.9] | d = 0.29 | 0.43 |
|  | *No-Stop HC vs. H&Y1* | t(86) = -2.99 | p = 0.058 | -100.0 | [-160.1, -40.0] | d = 0.98 | 24.51 |
|  | *No-Stop HC vs. H&Y2* | t(86) = -0.75 | p = 1 | -25.0 | [-93.5, 43.4] | d = 0.23 | 0.37 |
|  | *No-Stop HC vs. H&Y3* | t(86) = -0.02 | p = 1 | -0.8 | [-73.0, 71.5] | d = 0.01 | 0.29 |
|  | *No-Stop H&Y1 vs. H&Y2* | t(86) = 2.05 | p = 0.701 | 75.0 | [0.3, 149.8] | d = 0.64 | 1.53 |
|  | *No-Stop H&Y1 vs. H&Y3* | t(86) = 2.71 | p = 0.131 | 99.3 | [21.1, 177.4] | d = 0.81 | 3.84 |
|  | *No-Stop H&Y2 vs. H&Y3* | t(86) = 0.66 | p = 1 | 24.2 | [-60.2, 108.6] | d = 0.18 | 0.35 |
|  |  |  |  |  |  |  |  |
| **Three-way ANOVA on MT Between-Participants Factors: Group (H&Y1, H&Y2, H&Y3); Within-Participant Factors: Treatment (ON, OFF); Trial type (No-Stop, Go-Only)** | | | | | | | |
|  |  | **Value of parameters** | ***p-*values** | **M_diff_** | **95% CI** | **Effect Size** | **BF_10_** |
| Main effect: | **Group** | F[2,57] = 5.32 | **p = 0.008** | - | - | ηₚ² = 0.16 | 6.68 |
| *Post Hoc tests:* | *H&Y1 vs. H&Y2* | t(57) = -2.93 | **p = 0.015** | -150.4 | [-211.0, -89.8] | d = 0.78 | >100 |
|  | *H&Y1 vs. H&Y3* | t(57) = -2.70 | **p = 0.027** | -138.7 | [-195.2, -82.2] | d = 0.77 | >100 |
|  | *H&Y2 vs. H&Y3* | t(57) = 0.23 | p = 1 | 11.7 | [-51.2, 74.6] | d = 0.06 | 0.18 |
| Main effect: | **Treatment** | F[1,57] = 0.59 | p = 0.445 | -15.7 | [-47.3, 15.8] | ηₚ² = 0.01 | 0.22 |
| Main effect: | **Trial type** | F[1,57] = 5.43 | **p = 0.023** | -36.1 | [-61.7, -10.5] | ηₚ² = 0.09 | 1.57 |
| Interaction: | **Group x Treatment** | F[2,57] = 0.81 | p = 0.451 | - | - | ηₚ² = 0.03 | 0.24 |
| Interaction: | **Group x Trial type** | F[2,57] = 1.09 | p = 0.343 | - | - | ηₚ² = 0.04 | 0.21 |
| Interaction: | **Treatment x Trial type** | F[1,57] = 0.02 | p = 0.882 | - | - | ηₚ² < 0.001 | 0.19 |
| Interaction: | **Group x Treatment x Trial type** | F[2,57] = 0.35 | p = 0.708 | - | - | ηₚ² = 0.01 | 0.14 |
|  |  |  |  |  |  |  |  |
| **Two-way ANOVA on MT in OFF condition Between-Participants Factors: Group (HC, H&Y1, H&Y2, H&Y3) Within-Participant Factors: Trial type (No-Stop, Go-Only)** | | | | | | | |
|  |  | **Value of parameters** | ***p-*values** | **M_diff_** | **95% CI** | **Effect Size** | **BF_10_** |
| Main effect: | **Group** | F[3,86] = 8.99 | **p < 0.001** | - | - | ηₚ² = 0.24 | >100 |
| *Post Hoc tests:* | *HC vs. H&Y1* | t(86) = -0.65 | p = 1 | -30.0 | [-101.1, 41.1] | d = 0.18 | 0.31 |
|  | *HC vs. H&Y2* | t(86) = -4.45 | **p < 0.001** | -205.1 | [-279.3, -131] | d = 1.21 | >100 |
|  | *HC vs. H&Y3* | t(86) = -3.55 | **p = 0.004** | -163.8 | [-225.7, -101.9] | d = 1.09 | >100 |
|  | *H&Y1 vs. H&Y2* | t(86) = -3.47 | **p = 0.005** | -175.1 | [-263.2, -87.1] | d = 0.89 | >100 |
|  | *H&Y1 vs. H&Y3* | t(86) = -2.65 | p = 0.058 | -133.8 | [-212.0, -55.5] | d = 0.76 | 29.21 |
|  | *H&Y2 vs. H&Y3* | t(86) = 0.82 | p = 1 | 41.4 | [-39.7, 122.4] | d = 0.23 | 0.36 |
| Main effect: | **Trial type** | F[1,86] = 10.25 | **p = 0.002** | -44.7 | [-74.3, -19.8] | ηₚ² = 0.11 | 26.7 |
| Interaction: | **Group x Trial type** | F[3,86] = 0.91 | p = 0.442 | - | - | ηₚ² = 0.03 | 0.17 |
|  |  |  |  |  |  |  |  |
| **Two-way ANOVA on MT in ON condition Between-Participants Factors: Group (HC, H&Y1, H&Y2, H&Y3) Within-Participant Factors: Trial type (No-Stop, Go-Only)** | | | | | | | |
|  |  | **Value of parameters** | ***p-*values** | **M_diff_** | **95% CI** | **Effect Size** | **BF_10_** |
| Main effect: | **Group** | F[3,86] = 5.86 | **p < 0.001** | - | - | ηₚ² = 0.17 | 32.3 |
| *Post Hoc tests:* | *HC vs. H&Y1* | t(86) = -0.55 | p = 1 | -27.5 | [-88.2, 33.3] | d = 0.19 | 0.31 |
|  | *HC vs. H&Y2* | t(86) = -3.08 | **p = 0.016** | -153.1 | [-232.6, -73.6] | d = 0.85 | >100 |
|  | *HC vs. H&Y3* | t(86) = -3.45 | **p = 0.005** | -171.1 | [-249.3, -92.9] | d = 0.97 | >100 |
|  | *H&Y1 vs. H&Y2* | t(86) = -2.31 | p = 0.139 | -125.6 | [-210.8, -40.5] | d = 0.66 | 8.99 |
|  | *H&Y1 vs. H&Y3* | t(86) = -2.64 | p = 0.059 | -143.7 | [-227.5, -59.8] | d = 0.76 | 30.05 |
|  | *H&Y2 vs. H&Y3* | t(86) = -0.33 | p = 1 | -18.0 | [-115.9, 79.9] | d = 0.08 | 0.25 |
| Main effect: | **Trial type** | F[1,86] = 9.58 | **p = 0.003** | -42.5 | [-72.0, -18.1] | ηₚ² = 0.10 | 20.0 |
| Interaction: | **Group x Trial type** | F[3,86] = 1.23 | p = 0.304 | - | - | ηₚ² = 0.04 | 0.24 |

Statistically significant results are reported in bold. All Post hoc tests (pairwise comparisons) had an adjusted alpha level corrected according to Bonferroni. Bayes factors report the ratio between the null versus the alternative hypothesis (BF10). Effect sizes are reported as partial eta squared (ηp2) and Cohen's d. Abbreviations. Analysis of variance (ANOVA). Confidence Interval (CI), Hoehn & Yahr (H&Y) groups 1-1.5, 2, and 2.5-3 (H&Y1, H&Y2, and H&Y3, respectively). Assessment performed under pharmacological treatment (ON) and after an overnight wash-out of at least 12 hours (OFF).

## References

1. Obeso I, Wilkinson L, Jahanshahi M. Levodopa medication does not influence motor inhibition or conflict resolution in a conditional stop-signal task in Parkinson's disease. *Exp Brain Res*. Sep 2011;213(4):435-45. doi:10.1007/s00221-011-2793-x

2. Alegre M, Lopez-Azcarate J, Obeso I*, et al*. The subthalamic nucleus is involved in successful inhibition in the stop-signal task: a local field potential study in Parkinson's disease. *Exp Neurol*. Jan 2013;239:1-12. doi:10.1016/j.expneurol.2012.08.027

3. George JS, Strunk J, Mak-McCully R, Houser M, Poizner H, Aron AR. Dopaminergic therapy in Parkinson's disease decreases cortical beta band coherence in the resting state and increases cortical beta band power during executive control. *Neuroimage Clin*. 2013;3:261-70. doi:10.1016/j.nicl.2013.07.013

4. Cerasa A, Donzuso G, Morelli M*, et al*. The motor inhibition system in Parkinson's disease with levodopa-induced dyskinesias. *Mov Disord*. Dec 2015;30(14):1912-20. doi:10.1002/mds.26378

5. Picazio S, Ponzo V, Caltagirone C, Brusa L, Koch G. Dysfunctional inhibitory control in Parkinson's disease patients with levodopa-induced dyskinesias. *J Neurol*. Sep 2018;265(9):2088-2096. doi:10.1007/s00415-018-8945-1

6. Wylie SA, van Wouwe NC, Godfrey SG*, et al*. Dopaminergic medication shifts the balance between going and stopping in Parkinson's disease. *Neuropsychologia*. Jan 31 2018;109:262-269. doi:10.1016/j.neuropsychologia.2017.12.032

7. Manza P, Schwartz G, Masson M*, et al*. Levodopa improves response inhibition and enhances striatal activation in early-stage Parkinson's disease. *Neurobiol Aging*. Jun 2018;66:12-22. doi:10.1016/j.neurobiolaging.2018.02.003

8. Choudhury S, Roy A, Mondal B*, et al*. Slowed Movement Stopping in Parkinson's Disease and Focal Dystonia is Improved by Standard Treatment. *Sci Rep*. Dec 20 2019;9(1):19504. doi:10.1038/s41598-019-55321-5

9. Claassen DO, van den Wildenberg WP, Harrison MB*, et al*. Proficient motor impulse control in Parkinson disease patients with impulsive and compulsive behaviors. *Pharmacol Biochem Behav*. Feb 2015;129:19-25. doi:10.1016/j.pbb.2014.11.017

10. Logan GD, Cowan WB, Davis KA. On the ability to inhibit simple and choice reaction time responses: a model and a method. *J Exp Psychol Hum Percept Perform*. Apr 1984;10(2):276-91.

11. Band GP, van der Molen MW, Logan GD. Horse-race model simulations of the stop-signal procedure. *Acta Psychol (Amst)*. Feb 2003;112(2):105-42.

12. Logan GD. On the ability to inhibit thought and action: A users' guide to the stop signal paradigm. In: Inhibitory Processes in Attention, Memory and Language (Eds. Dagenbach, D., Carr, T. H.) *Academic Press, San Diego*. 1994;

13. Verbruggen F, Aron AR, Band GP*, et al*. A consensus guide to capturing the ability to inhibit actions and impulsive behaviors in the stop-signal task. *Elife*. Apr 29 2019;8doi:10.7554/eLife.46323

14. Levitt S, Gutin B. Multiple choice reaction time and movement time during physical exertion. *Res Q*. Dec 1971;42(4):405-10.

15. Verbruggen F, Chambers CD, Logan GD. Fictitious inhibitory differences: how skewness and slowing distort the estimation of stopping latencies. *Psychol Sci*. Mar 1 2013;24(3):352-62. doi:10.1177/0956797612457390

16. Boucher L, Palmeri TJ, Logan GD, Schall JD. Inhibitory control in mind and brain: an interactive race model of countermanding saccades. *Psychol Rev*. Apr 2007;114(2):376-97. doi:10.1037/0033-295X.114.2.376

17. Congdon E, Mumford JA, Cohen JR, Galvan A, Canli T, Poldrack RA. Measurement and reliability of response inhibition. *Front Psychol*. 2012;3:37. doi:10.3389/fpsyg.2012.00037
